# Whole-exome sequencing identifies *ECPAS* as a novel potentially pathogenic gene in multiple hereditary families with nonsyndromic orofacial cleft

**DOI:** 10.1093/procel/pwae021

**Published:** 2024-05-02

**Authors:** Huaxiang Zhao, Wenjie Zhong, Wenbin Huang, Guozhu Ning, Jieni Zhang, Mengqi Zhang, Peiqi Meng, Yunfan Zhang, Qian Zhang, Hongping Zhu, Gulibaha Maimaitili, Yi Ding, Weiran Li, Wei Liang, Zhibo Zhou, Qiang Wang, Feng Chen, Jiuxiang Lin

**Affiliations:** Department of Orthodontics, Peking University School and Hospital of Stomatology, Beijing 100081, China; Key Laboratory of Shaanxi Province for Craniofacial Precision Medicine Research, College of Stomatology, Xi’an Jiaotong University, Xi’an 710049, China; Department of Orthodontics, Peking University School and Hospital of Stomatology, Beijing 100081, China; College of Stomatology, Chongqing Medical University; Chongqing Key Laboratory of Oral Diseases and Biomedical Sciences; Chongqing Municipal Key Laboratory of Oral Biomedical Engineering of Higher Education, Chongqing 400016, China; Department of Orthodontics, Peking University School and Hospital of Stomatology, Beijing 100081, China; Department of Orthodontics, Stomatological Center, Peking University Shenzhen Hospital, Shenzhen Peking University-The Hong Kong University of Science and Technology Medical Center, Shenzhen 518036, China; Affiliated Hospital of Guangdong Medical University & Zhanjiang Key Laboratory of Zebrafish Model for Development and Disease, Guangdong Medical University, Zhanjiang 524023, China; Department of Orthodontics, Peking University School and Hospital of Stomatology, Beijing 100081, China; National Center of Stomatology, National Clinical Research Center for Oral Diseases, National Engineering Laboratory for Digital and Material Technology of Stomatology, Beijing Key Laboratory for Digital Stomatology, Research Center of Engineering and Technology for Computerized Dentistry Ministry of Health, NMPA Key Laboratory for Dental Materials, Beijing 100191, China; Department of Orthodontics, Peking University School and Hospital of Stomatology, Beijing 100081, China; Department of Orthodontics, Peking University School and Hospital of Stomatology, Beijing 100081, China; Department of Orthodontics, Peking University School and Hospital of Stomatology, Beijing 100081, China; National Center of Stomatology, National Clinical Research Center for Oral Diseases, National Engineering Laboratory for Digital and Material Technology of Stomatology, Beijing Key Laboratory for Digital Stomatology, Research Center of Engineering and Technology for Computerized Dentistry Ministry of Health, NMPA Key Laboratory for Dental Materials, Beijing 100191, China; Central Laboratory, Peking University School and Hospital of Stomatology, Beijing 100081, China; National Center of Stomatology, National Clinical Research Center for Oral Diseases, National Engineering Laboratory for Digital and Material Technology of Stomatology, Beijing Key Laboratory for Digital Stomatology, Research Center of Engineering and Technology for Computerized Dentistry Ministry of Health, NMPA Key Laboratory for Dental Materials, Beijing 100191, China; Department of Oral and Maxillofacial Surgery, Peking University School and Hospital of Stomatology, Beijing 100191, China; The Second Affiliated Hospital of Xinjiang Medical University and Xinjiang Key Laboratory of Neurological Disorder Research, Urumqi 830028, China; Department of Physiology and Pathophysiology, School of Basic Medical Sciences, Xi’an Jiaotong University, Xi’an 710049, China; Department of Orthodontics, Peking University School and Hospital of Stomatology, Beijing 100081, China; National Center of Stomatology, National Clinical Research Center for Oral Diseases, National Engineering Laboratory for Digital and Material Technology of Stomatology, Beijing Key Laboratory for Digital Stomatology, Research Center of Engineering and Technology for Computerized Dentistry Ministry of Health, NMPA Key Laboratory for Dental Materials, Beijing 100191, China; Department of Orthodontics, Peking University School and Hospital of Stomatology, Beijing 100081, China; National Center of Stomatology, National Clinical Research Center for Oral Diseases, National Engineering Laboratory for Digital and Material Technology of Stomatology, Beijing Key Laboratory for Digital Stomatology, Research Center of Engineering and Technology for Computerized Dentistry Ministry of Health, NMPA Key Laboratory for Dental Materials, Beijing 100191, China; National Center of Stomatology, National Clinical Research Center for Oral Diseases, National Engineering Laboratory for Digital and Material Technology of Stomatology, Beijing Key Laboratory for Digital Stomatology, Research Center of Engineering and Technology for Computerized Dentistry Ministry of Health, NMPA Key Laboratory for Dental Materials, Beijing 100191, China; Department of Oral and Maxillofacial Surgery, Peking University School and Hospital of Stomatology, Beijing 100191, China; Innovation Centre of Ministry of Education for Development and Diseases, Sixth Affiliated Hospital, School of Medicine, South China University of Technology, Guangzhou 511442, China; National Center of Stomatology, National Clinical Research Center for Oral Diseases, National Engineering Laboratory for Digital and Material Technology of Stomatology, Beijing Key Laboratory for Digital Stomatology, Research Center of Engineering and Technology for Computerized Dentistry Ministry of Health, NMPA Key Laboratory for Dental Materials, Beijing 100191, China; Central Laboratory, Peking University School and Hospital of Stomatology, Beijing 100081, China; Department of Orthodontics, Peking University School and Hospital of Stomatology, Beijing 100081, China; National Center of Stomatology, National Clinical Research Center for Oral Diseases, National Engineering Laboratory for Digital and Material Technology of Stomatology, Beijing Key Laboratory for Digital Stomatology, Research Center of Engineering and Technology for Computerized Dentistry Ministry of Health, NMPA Key Laboratory for Dental Materials, Beijing 100191, China

**Dear Editor,**

Orofacial cleft (OFC), which includes cleft lip and/or palate (CL/P) and cleft palate (CP), is the most common congenital craniofacial structural disorder, with a prevalence of 1.416‰ among live infants worldwide (Massenburg et al., 2021). Nonsyndromic OFC (NSOFC), which does not contain other malformations as syndromic OFC (SOFC), accounts for 70% of cases and is believed to have complex etiologies. Notably, it has been established that genetic factors play a crucial role in the occurrence of NSOFC (Dixon et al., 2011).

Prior to the comprehensive analysis of the entire human genome, linkage analysis, and candidate gene association studies had been the predominant approaches for exploring the genetic basis of NSOFC. Along with advances in genetics, genome-wide association studies (GWAS) are capable of identifying risk loci associated with NSOFC across the entire genome, independently of predetermined candidate genes. Up to now, GWAS have unveiled more than 45 genetic risk loci, usually with minor allele frequency (MAF) higher than 5%, including 1q32 (*IRF6*), 3q27 (*TP63*), 9q (*FOXE1*), etc., that collectively account for 10%–30% of the heritability of NSOFC (Leslie, 2022). However, there is still a significant proportion of genetic contribution to NSOFC that remains unexplained, limiting the diagnostic utility of clinical genetic testing, as is the case with other congenital deformities (Lord et al., 2019).

The implementation of next-generation sequencing strategies and functional studies has markedly advanced the identification of genetic variants associated with NSOFCs. Previous studies have indicated the substantial role of *de novo* variants (DNVs) or rare variants within clinically relevant OFC genes such as *PTCH1*, *GRHL3*, and *CTNND1*, which significantly contributed to the genetic etiology of NSOFC (He et al., 2022; Huang et al., 2023; Leslie, 2022). While DNVs are more important in sporadic cases, rare variants appear to play a greater role in hereditary pedigrees (Bishop et al., 2020; Zuk et al., 2014). Therefore, a systematic study of rare variants in patients from hereditary families is likely to unveil causal genes for NSOFC.

The development of the lip and palate is a conserved process across vertebrates and is controlled by several key signaling pathways such as Hedgehog (HH), WNT, TGF-β, and FGF. Although previous studies have indicated that structural clefts can result from disturbances in these pathways (Reynolds et al., 2020), a comprehensive understanding of how dysregulation in these pathways contributes to NSOFC remains elusive.

We recruited 30 families with NSOFC over the past seven years, in which at least two members in each family were affected (Fig. S1). Among these families, 23 exhibited autosomal dominant (AD) inheritance and seven exhibited autosomal recessive (AR) inheritance (Fig. S1). To uncover rare variants that may confer the risk of NSOFC, we conducted whole-exome sequencing (WES) on these families. After applying filtering criteria, we identified a total of 394 candidate variants related to OFC/craniofacial development in these 30 hereditary families (Fig. 1A; Table S1), and delineated the genetic architecture (Fig. S2). Next, we turned our attention to those pathogenic/likely pathogenic variants known to have roles in the OFC-related morphogenic processes or pathways. Following the American College of Medical Genetics and Genomics (ACMG) criteria, we identified nine pathogenic/likely pathogenic variants from seven out of the 30 families (23.33%) (Fig. 1B and 1C; Table S2). These variants included *PTCH1* (two families, 6.67%), *GLI2* (one family, 3.33%), *IRF6* (one family, 3.33%), *PLEKHA5* (one family, 3.33%), *CREBBP* (one family, 3.33%), and *FZD6* (one family, 3.33%) (Fig. 1B). These variants fell into four morphogenic pathways crucial for lip and palate development: HH (three families, 10.00%), epithelial-related (two families, 6.67%), TGF-β (one family, 3.33%), and WNT (one family, 3.33%) signaling pathway (Fig. 1C), confirming the critical roles of these pathways for the pathogenesis of OFC and validating the reliability of our data. Apart from these known causal variants, we also detected many novel variants, which, without known functions for either lip/palate development or pathogenesis of OFC, represent a rich resource for future research.

**Figure 1.**
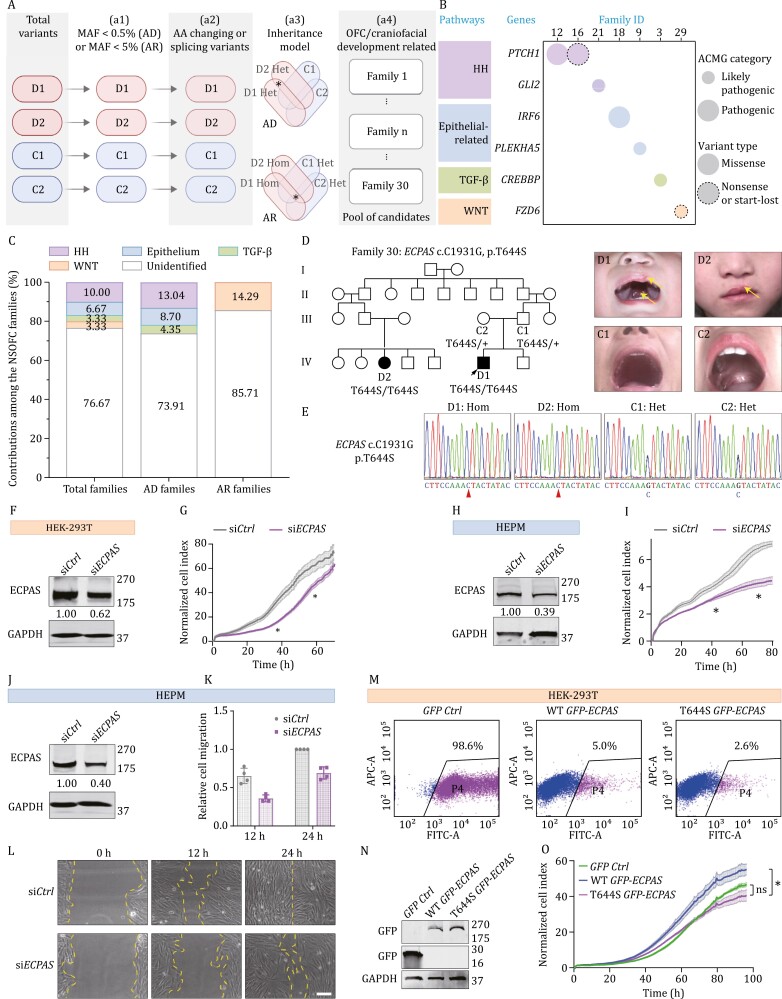
**Identification of T644S variant of *ECPAS* in an AR-inheritance family with NSOFC and ECPAS T644S variant displaying impaired capacity to promote cell proliferation.** (A) Screening process to identify candidate rare variants in hereditary families with NSOFC: (a1) variants with MAF higher than 0.5% were excluded for pedigrees with AD inheritance, while variants with MAF higher than 5% were excluded for pedigrees with AR inheritance; (a2) inclusion of variants causing amino acid changes (missense, nonsense, insertion, deletion, etc.) or splicing variants; (a3) the most appropriate Mendelian inheritance model was applied to further narrow down the candidate variants, including heterozygous variants carried by patients but absent in unaffected family members for families with AD inheritance, and homozygous variants carried by patients along with heterozygous variants present in unaffected family members for families with AR inheritance; (a4) a self-developed web crawler and the Phenolyzer software were employed to identify genes associated with OFC or craniofacial development. (B) Distribution and (C) contribution of known causal genes in morphogenic processes or pathways related to lip and palate development in multiple hereditary families. The size of the circles represents the classification of variants according to the ACMG guidelines, with small circles indicating likely pathogenic variants and large circles indicating pathogenic variants. Solid circles denote missense variants, while dashed circles represent nonsense or start-loss variants. HH, Hedgehog pathway; Epithelial-related, epithelial-related pathway; TGF-β, TGF-β pathway; WNT, WNT pathway. Detailed information of variants is listed in Table S2. (D) The pedigree and clinical images of the proband (D1), his affected cousin (D2) and his unaffected parents (C1 and C2) in Family 30. The arrow in the pedigree map indicates the proband and arrows in the photographs indicate the regions affected by clefts. (E) Sanger sequencing chromatograms of persons shown in (D). Primers for PCR-Sanger sequencing of ECPAS T644S variant: F-5ʹ-CCCAAGTGAAAGCAA-3ʹ, R-5ʹ-AACCCAACAAGGAGG-3ʹ. Arrowheads indicate the site of nucleotide substitution. Hom, homozygous variant; Het, heterozygous variant. (F–I) HEK-293T and HEPM cells transfected with negative control siRNA (si*Ctrl*) or *ECPAS*-targeting siRNA (si*ECPAS*) were subjected to real-time proliferation assays. ECPAS knockdown significantly inhibited the proliferation of both HEK-293T (epithelial) and HEPM (mesenchymal) cells. (J–L) HEPM cells transfected with si*Ctrl* or si*ECPAS* were processed for a wound healing assay. ECPAS knockdown inhibited the migratory capacity of HEPM cells. (M–O) HEK-293T cells transfected with GFP control, wild-type (WT) GFP-ECPAS or T644S GFP-ECPAS constructs were sorted with flow cytometry, followed by real-time proliferation assays. Overexpression of WT ECPAS, but not T644S ECPAS, enhanced cell proliferation compared to the GFP control (at 60 h and 80 h). Notably, while the cell number after the overexpression of the ECPAS T644S mutant appeared slightly decreased compared to the GFP control, this difference did not reach statistical significance. The normalized cell index was presented as mean ± SD. Statistical analysis was performed using Student’s *t*-test or one-way AVONA. *P < 0.05; ns, not significant.

Noticing that no known pathogenic variants were detected in the majority of hereditary families exhibiting AR inheritance, we, therefore, focused on these families. In Family 30, a rare homozygous missense variant in the *ECPAS* gene (c.C1931G, p.T644S) came to our attention (Figs. 1D and S3). The proband (D1), a boy with left cleft lip (CL) and median CP, and one of his second cousins (D2), a girl with left CL, are the two individuals diagnosed with NSOFC in this four-generation family (Fig. 1D). Using PCR-Sanger sequencing, we confirmed an AR inheritance model in this family (Fig. 1E). Then, we conducted *in silico* analysis to evaluate the impact of T644S on the function of ECPAS, which suggested that T644S variant is possibly pathogenic (Fig. S4).

*ECPAS*, also known as *ECM29*, is a proteasome-associated protein that plays an important role in cell proliferation and migration (Gorbea et al., 2004; Miettinen et al., 2018). Considering the coordinated proliferation and migration of epithelial and mesenchymal cells are essential for craniofacial morphogenesis, we next assessed whether ECPAS might affect cell proliferation and migration and whether the T664S variant might alter this functional role. As expected, the knockdown of *ECPAS* significantly inhibited cell proliferation in both HEK-293T (epithelial) and HEPM (mesenchymal) cells (Fig. 1F–I). Besides, the knockdown of *ECPAS* also reduced the migratory capacity of HEPM cells (Fig. 1J–L). Conversely, overexpression of wild-type ECPAS significantly promoted cell proliferation in HEK-293T cells (Fig. 1M–O). In contrast, although overexpression of ECPAS T664S appeared to slightly inhibit cell proliferation compared to the GFP control, this difference was not statistically significant, indicating that overexpressed ECPAS T644S mutant behaved like the GFP control (Fig. 1M–O). These results corroborate the role of ECPAS in boosting cell proliferation and migration as previously reported (Miettinen et al., 2018), and the bi-allelic T644S variant is a loss-of-function mutation that might be pathogenic for NSOFC.

We notice that the *Ecpas*-null mice exhibited impaired disassembly of the 26S proteasome under oxidative stress conditions and no craniofacial defect has been observed (Haratake et al., 2016). However, since the penetrance of clefts in knockout mice models is typically low and can sometimes be neglected (Peyrard-Janvid et al., 2014), a potential connection between ECPAS and craniofacial development cannot be entirely ruled out. We examined the spatiotemporal expression pattern of ECPAS in mice from E11.5 to E16.5. Using qPCR and immunohistochemistry (IHC), we found that ECPAS expression increased steadily during lip development (Fig. S5), suggesting a possible involvement of ECPAS in mouse lip development.

To determine whether ECPAS plays a role in craniofacial development, we turned to the zebrafish model. We first employed *in situ* hybridization (ISH) to investigate the expression pattern of *ecpas* in zebrafish embryos, and our findings demonstrated that *ecpas* is expressed maternally at one and two-cell stages and later in the craniofacial region from 36 h postfertilization (hpf) to 3 days postfertilization (dpf) (Fig. S6). Next, we designed translation-blocking and splicing-blocking antisense morpholino oligonucleotides (MOs) targeting the zebrafish *ecpas* gene, which effectively and specifically inhibited the expression of Ecpas protein (Figs. S7 and S8A). Compared to the negative control MO (CMO), microinjection of *ecpas* ATG MO or *ecpas* splicing MO caused craniofacial dysplasia, characterized by a less protruding mouth, at 4 dpf (Figs. 2A and S8B). Notably, a similar phenotype was observed when *ecpas* MO was co-injected with *p53* MO, indicating that this phenotype was not due to nonspecific cytotoxicity mediated by the p53 pathway (Robu et al., 2007). We then performed Alcian blue staining to visualize the craniofacial cartilage. In comparison to embryos injected with control MO, *ecpas* morphants had significantly smaller ethmoid plates and palatoquadrates. Moreover, the depletion of *ecpas* also led to a reduction in the size of Meckel’s cartilages, as well as developmental abnormalities in the rest of the pharyngeal region, including shortened arches and enlarged arch angles (Figs. 2B, S8C and S9).

**Figure 2.**
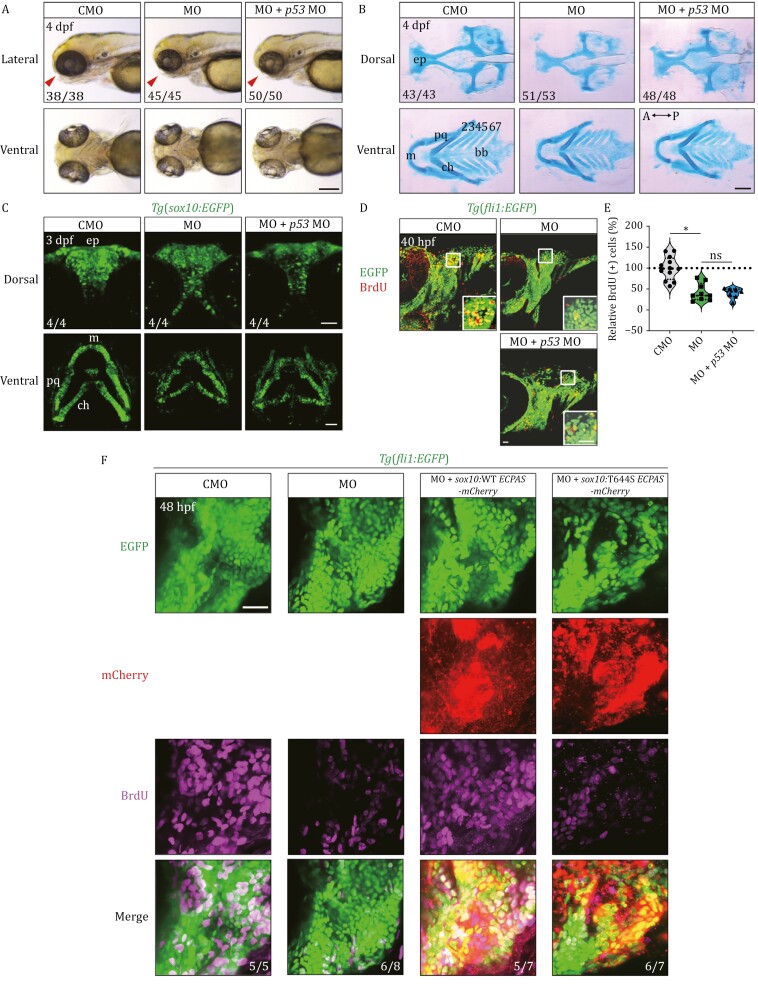
**Loss of *ecpas* impairs craniofacial development in zebrafish by inhibiting the proliferation of CNCCs, and human wild-type *ECPAS*, but not the T644S variant, rescues *ecpas* MO-induced impaired CNCC proliferation in zebrafish embryos.** (A) Lateral and ventral views of zebrafish head morphology at 4 dpf. Note that microinjection of *ecpas* MO (MO), but not control MO (CMO) led to craniofacial dysplasia characterized by a less protruding mouth. The mouth region was indicated by arrowheads. Scale bar, 200 μm. (B) Dorsal and ventral views of head cartilages stained with Alcian blue at 4 dpf. Zebrafish embryos injected with *ecpas* MO exhibited significantly smaller head cartilages, such as ethmoid plates, palatoquadrates and Meckel’s cartilages. ep, ethmoid plate; m, Meckel’s; pq, palatoquadrate; ch, ceratohyal; bb, basibranchial; 2–7, the second to seventh pharyngeal arches. Scale bar, 100 μm. (C) Dorsal and ventral views of *Tg*(*sox10*:*EGFP*) transgenic zebrafish embryos with CNCCs labelled with GFP. Knockdown of *ecpas* resulted in a reduction in the number of CNCCs in the ethmoid plate, Meckel’s cartilage and palatoquadrate cartilage at 3 dpf. Scale bar, 50 μm. (D and E) Confocal images showing BrdU-positive cells (red) in *Tg*(*fli1*:*EGFP*) transgenic zebrafish embryos with CNCCs labeled with GFP at 40 hpf. Note a significant decrease in the ratio of BrdU-positive cells in the pharyngeal region of *ecpas* morphants. Scale bar, 50 μm (20 μm in the magnified view). (F) Confocal images showing BrdU-positive cells (magenta channel) within the first and second pharyngeal arches (PA1–2) region of *Tg*(*fli1*:*EGFP*) transgenic zebrafish embryos at 48 hpf. The CNCCs were labeled with GFP (green channel). A noticeable reduction in BrdU-positive cells was observed in the *ecpas* MO group (magenta channel in second column), compared to the CMO group (magenta channel in first column). *sox10* promoter-driven CNCC-specific overexpression of wild-type *ECPAS* (red channel in third column) rescued the proliferation impaired by *ecpas* MO (magenta channel in third column). In contrast, the T644S variant, despite being overexpressed at levels comparable to the wild-type (red channel in fourth column), did not reverse the *ecpas* MO-induced proliferation deficit of CNCCs (magenta channel in fourth column). Scale bar, 20 μm. *sox10*:*WT*/*T644S ECPAS-mCherry*, the *sox10* promoter-driven recombinant Tol2 vector containing either human WT or T644S *ECPAS* cDNA, fused with a mCherry tag at the C-terminal. CNCCs, cranial neural crest cells. CMO, the negative control MO; MO, MO targeting *ecpas* ATG; *p53* MO, MO targeting *p53*. Hpf, hours postfertilization; Dpf, days postfertilization. The ratios of embryos with representative phenotypes were indicated. Data represent mean ± SD. One-way AVONA for statistical analysis. *P < 0.05; ns, not significant.

As craniofacial cartilage originates from cranial neural crest cells (CNCCs) (Cordero et al., 2011) and ECPAS promotes cell proliferation and migration, we sought to determine the effect of *ecpas* loss on these cells. In *Tg*(*sox10*:*EGFP*) transgenic zebrafish embryos, which enabled us to visualize CNCCs with GFP fluorescence, we observed that knockdown of *ecpas* led to a reduction in the number of CNCCs in the ethmoid plate, Meckel’s and palatoquadrate cartilages at 3 dpf (Fig. 2C). We next conducted a BrdU assay to assess the proliferative status of CNCCs. Immunofluorescence analysis revealed a significant decrease in the ratio of BrdU-positive cells in the pharyngeal region of *ecpas* morphants at 40 hpf (Fig. 2D and 2E), indicating impaired proliferation of CNCCs upon *ecpas* loss. However, except for the maternal expression of *ecpas* (Fig. S6), we did not observe any effect of *ecpas* disruption on the early stage of CNCCs migration at 24 hpf (Fig. S10A), or the later stage of CNCCs migration into the pharyngeal region at 36 hpf (Fig. S10B).

To examine whether the T644S variant of *ECPAS* has a loss-of-function effect on the proliferation of CNCCs in zebrafish embryos, akin to its role observed in cellular experiments, we performed a rescue experiment in zebrafish embryos. *Tg*(*fli1*:*EGFP*) transgenic zebrafish embryos, where CNCCs and blood vessels are labeled with GFP, were used in this experiment. We co-injected a *sox10* promoter-driven recombinant Tol2 vector containing either the wild-type or T644S variant of human *ECPAS* tagged with mCherry at its C-terminal and transposase mRNA to ensure CNCCs-specific overexpression of these two constructs. We observed that overexpression of wild-type ECPAS could rescue the impaired proliferation of CNCCs induced by *ecpas* MO. However, the T644S variant, despite being overexpressed at levels comparable to the wild-type, failed to rescue the *ecpas* MO-induced weakened proliferation of CNCCs in zebrafish embryos (Fig. 2F). These results suggest that disruption of *ecpas* impairs craniofacial development in zebrafish by inhibiting the proliferation of CNCCs and the T644S variant is indeed a loss-of-function mutation that impairs the proliferation-promoting capacity of *ECPAS in vivo*.

In this study, we made three main findings. First, we delineate the genetic architecture in patients with NSOFC and are able to uncover many novel candidate variants by WES in multiple Chinese hereditary families. Second, we identified a bi-allelic loss-of-function variant in the *ECPAS* gene, T644S, in a hereditary NSOFC family showing an AR inheritance. Third, ECPAS promotes cell proliferation in mammalian cells and loss of *ecpas* impairs craniofacial development in zebrafish by inhibiting the proliferation of CNCCs.

Several limitations of this study should be mentioned. First, the use of MO in zebrafish presents challenges, such as incomplete suppression of gene expression, which limits the ability to observe phenotypes that require complete gene knockout or extended suppression. In addition, off-target effects can obscure the interpretation of MO-induced phenotypes, even with proper controls. In future studies, an effective CRISPR/Cas9 system could be utilized to create loss-of-function mutants, providing clearer links between the genotype and phenotype. Moreover, it’s important to recognize that when it comes to mimicking missense variants, knock in mouse models are more accurate compared to zebrafish models. Therefore, our future research will focus on establishing a knock in mouse model, to corroborate our conclusions and enhance the understanding of the underlying mechanisms.

## Supplementary data

Supplementary data is available at https://doi.org/10.1093/procel/pwae021.

pwae021_suppl_Supplementary_Figures_S1-S10_Tables_S1-S2

## Notes

This study was supported by grants from the National Nature Science Foundation of China (81870747, 81900984, and 82170916), the Fundamental Research Funds for the Central Universities (PKU2022XGK001), the Beijing Municipal Natural Science Foundation (7182184), the National Key Research and Development Program of China (2020YFA0804000 and 2016YFC1000804), and the Xinjiang Key Laboratory of Neurological Disorder Research Fund (XJDX1711-2213).

## Notes

All procedures followed were in accordance with the ethical standards of the responsible committee on human experimentation (PKUSSIRB-201520012, Ethical Committee of Peking University Hospital of Stomatology, China) and with the Helsinki Declaration of 1975, as revised in 2000 (5). Informed consent was obtained from all participants or their guardians for being included in the study.

All institutional and national guidelines for the care and use of laboratory animals were followed (LA2018192, Peking University Animal Ethics Committee, China).

## Notes

J.L., F.C., Q.W., Z.Z., and W.L. designed the research; H.Z., W.Z., W.H., G.N., J.Z., M.Z., P.M., Y.Z., Q.Z., H.Z., G.M., Y.D., and W.L. performed the research; J.L., F.C., Q.W., Z.Z., and W.L. analyzed the data; H.Z., W.Z., W.H., J.L., F.C., and Y.D. wrote the manuscript. H.Z., G.N., W.H., W.Z., J.Z., and Y.D. revised the manuscript.
