# Supplementary material for: Whole-exome sequencing identifies ECPAS as a novel potentially pathogenic gene in multiple hereditary families with nonsyndromic orofacial cleft
Source: Protein Cell. 2024 May 2;15(10):783–9. doi: 10.1093/procel/pwae021 (PMC11443446; doi:10.1093/procel/pwae021)
Supplement: pwae021_suppl_Supplementary_Figures_S1-S10_Tables_S1-S2 [file pwae021_suppl_supplementary_figures_s1-s10_tables_s1-s2.pdf]

Supplemental materials for

Whole-exome sequencing identifies *ECPAS* as a novel potentially pathogenic gene in multiple hereditary families with nonsyndromic orofacial cleft

Huaxiang Zhao[1, 2, #], Wenjie Zhong[1, 3, #], Wenbin Huang[1, 4, #], Guozhu Ning[5, #], Jieni Zhang[1, 6, #], Mengqi Zhang[1], Peiqi Meng[1], Yunfan Zhang[1], Qian Zhang[7, 6], Hongping Zhu[8, 6], Gulibaha Maimaitili[9], Yi Ding[10], Weiran Li[1, 6], Wei Liang[1, 6, *], Zhibo Zhou[8, 6, *], Qiang Wang[11, *], Feng Chen[7, 6, *], Jiuxiang Lin[1, 6, *]

# These authors contributed equally to this work.

* Correspondence: bmuliang@bjmu.edu.cn (Wei Liang), zzbooo@126.com (Zhibo Zhou), qiangwang@scut.edu.cn (Qiang Wang), chenfeng2011@hsc.pku.edu.cn (Feng Chen), and jxlin@pku.edu.cn (Jiuxiang Lin)

The supplemental materials include:

- ✧ **Materials and methods of this paper**
- ✧ **Figure S1-S10**
- ✧ **Table S1-S5**
- ✧ **References for the supplemental materials**

Materials and methods of this paper

(1) Ethics compliance

This research was approved by the Ethical Committee of Peking University Hospital of Stomatology (PKUSSIRB-201520012) and informed consent was obtained from the participants or their guardians.

All animal studies were approved by the Peking University Animal Ethics Committee (LA2018192), and the experiments were performed in strict accordance with the animal care.

(2) Clinical samples

Over the past seven years, we recruited 30 hereditary families with NSOFC, where at least two members were affected in each family. These families were from various ethnic groups in China, including 24 from the Han population, 4 from the Uighur population, one from the Hui population, and one from the Kazak population **(Table S3)**. Five of these families have been reported in previous studies (Families 12, 16, 18, 21 and 29) (Zhao et al., 2018a; Zhao et al., 2018b; Meng et al., 2019; Zhang et al., 2020; Zhong et al., 2020). To ensure a clear diagnosis of NSOFC, two associate chief surgeons examined all patients and excluded any other organ malformations except for OFC. We also examined accessible unaffected individuals in each family to rule out the possibility of occulting submucous OFC. Additionally, we recruited another independent cohort of unaffected subjects to confirm the allele frequency, as previously described (Zhao et al., 2018b). 2-4 mL of peripheral blood from each participant was collected, and genomic DNA was extracted using the QIAamp DNA Blood Mini Kit (Qiagen, #51106).

(3) WES and Sanger sequencing

WES was performed on the BGISEQ-500 platform (BGI Inc., China), and after quality control **(Table S4)**, the high-quality reads were mapped to the human

reference genome (GRCh37/HG19) following our previous methodology (Zhao et al., 2018b). The single-nucleotide variants (SNVs) and short insertion/deletion (InDels) were called and annotated using the GATK software (DePristo et al., 2011) and SnpEff tool (Cingolani et al., 2012), respectively **(Table S5)**. PCR-Sanger sequencing with specific primers was performed to validate WES results.

(4) Screening for disease-causing variants in each NSOFC hereditary family

All pedigrees were categorized into either AD or AR inheritance according to previous studies (McKusick, 1966a, b; Vikkula et al., 1995). Pedigrees were designated as AD inheritance when the affected probands had an affected parent, and cases of NSOFC appeared in successive generations. Conversely, AR inheritance was assigned to pedigrees where NSOFC patients did not exhibit a consecutive generational pattern. We first excluded variants with high frequency in the genomic database that are less likely to be pathogenic to NSOFC (Dixon et al., 2011). Specifically, we removed variants with MAF higher than 0.5% for pedigrees with AD inheritance, and MAF higher than 5% for pedigrees with AR inheritance, in accordance with our previous studies (Zhang et al., 2020) and the prevalence of OFC (Liu et al., 2021; Massenburg et al., 2021). We then focused on variants that cause amino acid changes (missense, nonsense, insertion, deletion, *etc.*) or splicing variants, which are more likely to be deleterious than non-coding variants (Huang et al., 2023a). Next, we employed the most appropriate Mendelian inheritance model to further narrow down the candidate variants. Specifically, 23 families exhibit autosomal dominant inheritance, and seven families exhibit autosomal recessive inheritance. To identify genes related to OFC or craniofacial development, a self-developed web crawler (Zhong et al., 2020) and the Phenolyzer software (Yang et al., 2015) were utilized. Finally, we manually annotated the pathogenicity of the remaining variants according to the ACMG guidelines (Richards et al., 2015; Tavtigian et al., 2020), referring in part to the VarSome Platform (v11.9) (Kopanos et al., 2019).

(5) Cell culture and transfection

The human embryonic kidney epithelial cells (HEK-293T) and the human embryonic palatal mesenchymal cells (HEPM) were purchased from the China National Infrastructure of Cell Line Resource and ATCC bank, respectively. The cells were cultured in DMEM medium, supplemented with 10% fetal bovine serum (Gibco, #10270-106) and 1% penicillin-streptomycin, in a 5% CO_2 incubator at 37 ℃. To introduce plasmids or siRNA into cells, the Lipo3000 reagent (Thermo, #L3000015) and electroporation (Celetrix, USA) were utilized according to the operation manual.

(6) Cloning

The full-length wild-type *ECPAS* coding sequence (Wang et al., 2017) was inserted into pCS2 vectors with a N-terminal GFP tag, and the T644S mutant was generated using site-directed mutagenesis. For the rescue experiment in zebrafish embryos, the cDNAs of either wild-type or T644S *ECPAS* were inserted into a *sox10* promoter-driven recombinant Tol2 vector, with a mCherry tag at the C-terminal (named *sox10:WT/T644S ECPAS-mCherry*) (Dutton et al., 2008; Takeuchi et al., 2010; Rodrigues et al., 2012; Rezaei et al., 2019). The sequences of all constructs used in this study were confirmed through directed Sanger sequencing.

(7) Proliferation and migration assay *in vitro*

iCELLigence real-time cell analysis system (ACE company, USA) was used to assess the capacity of cell proliferation and wound healing assay was performed to evaluate their migratory capacity *in vitro*.

(8) Western blot

The cell lysate was prepared for Western blot as described previously (Huang et al., 2023a). The antibodies used were: GFP mouse mAb (CST, #2955), GAPDH rabbit mAb (CST, #2118) and ECPAS rabbit pAb (Novus Biologicals, #NB100-74407). The lysate from zebrafish embryos was prepared for Western blot following previous studies (Ning et al., 2013). The antibodies used were: ECPAS rabbit pAb (Invitrogen, #PA5-82770) and β-Tubulin (CWBIO, #CW0098).

(9) Zebrafish

The wild-type embryos used in this study were Tübingen (TU) strains purchased from the China Zebrafish Resource Center. To visualize CNCCs, we used *Tg(sox10:EGFP)* and *Tg(sox10:mCherry-CAAX)* transgenic embryos, which express GFP and Cherry under the control of the *sox10* promoter (Dutton et al., 2008). In addition, we utilized *Tg(fli1:EGFP)* transgenic embryos that express GFP in CNCCs and blood vessels (Lawson and Weinstein, 2002). All zebrafish embryos were raised in Holtfreter's solution at 28.5℃ and were staged according to morphological characteristics as described (Kimmel et al., 1995).

(10) siRNA synthesis and MOs

To knockdown human *ECPAS in vitro*, siRNA oligos were synthesized by GenePharma Co. (Shanghai, China) based on the sequence obtained from previous studies (Wang et al., 2017). In order to knock down the zebrafish *ecpas*, MOs were designed and synthesized by Gene Tools. The primary MO used in our zebrafish experiments targets the translation initiation site of *ecpas* (named MO or *ecpas* ATG MO), with the sequence 5'-CGCAGCCATGTTTGAAGTGAAGTCA-3'. To rule out the possibility of non-specific phenotypic effects, we synthesized an addition MO, targeting the splicing region between the first exon and the first intron of the zebrafish *ecpas* gene (named *ecpas* splicing MO), with the sequences 5'-ATAATGAGTTAAACTTACTGAGCTC-3'. Additionally, negative control MOs (named CMO) and blocking zebrafish endogenous *p53* MOs (named *p53* MO) were utilized, as previously described (Ning et al., 2013).

(11) Alcian blue staining

Zebrafish embryos were cultured until 4 dpf, and then fixed overnight with 4% paraformaldehyde (PFA), followed by staining with 0.1 mg/ml Alcian blue reagent (Sigma, #5268). After destaining, the embryos were fixed with glycerin (Ning et al., 2013). We followed the previous methodology (Mork and Crump, 2015) to measure

the lengths and angles of zebrafish embryos' cartilage.

(12) BrdU labeling assay

We conducted BrdU labeling assay on *Tg(fli1:EGFP)* zebrafish embryos by treating them with 10 mM BrdU for 25 minutes at 40 hpf. Whole-mount immunostaining was performed using anti-BrdU (Sigma, #B2531) and anti-GFP (Invitrogen, #A-11122) antibodies as our previous work (Ning et al., 2013).

(13) ISH

As described previously (Ning et al., 2013), zebrafish embryos were fixed with 4% PFA and subjected to *in situ* hybridization with specific probes. The *dlx2* probe, known for labeling early CNCCs, was kindly provided by Anming Meng's Lab at Tsinghua University. For the *ecpas* probe, we amplified a fragment of 497 bp from the cDNA of 48-hpf embryos using the following primers: F primer (5'-GCTGGATAAGGAACATCAGAACGCT-3') and R primer (5'-GAAGTGCATACATGAGCAGTTGGAG-3'). The PCR product was subsequently purified using the PureLink purification kit (Invitrogen, #K3100-01) and served as the template for antisense probe synthesis, following the described method (Zhang et al., 2021). After blocking, the embryos were incubated with anti-Dig-AP and then stained using the BM purple AP substrate.

(14) IHC

Heads of embryonic mice from the ICR strain were fixed in 4% PFA and prepared as sagittal sections in paraffin (4 μm). IHC was conducted using the ECPAS rabbit pAb (Invitrogen, #PA5-82770) and HRP-conjugated anti-rabbit antibody (Promega, #W401B), following the procedures in our previous studies (Huang et al., 2023b).

(15) Zebrafish embryo rescue assay

Tg(fli1:EGFP) transgenic zebrafish embryos at the one-cell stage were microinjected with various combinations: (1) CMO, (2) *ecpas* MO, (3) *ecpas* MO + *sox10:WT*

ECPAS-mCherry vector + transposase mRNA, or (4) *ecpas* MO + *sox10:T644S ECPAS-mCherry* vector + transposase mRNA. The empty vector and mRNA served as negative controls for standardization across different groups. At 48 hpf, we conducted a BrdU assay to assess the proliferative status of cells, as detailed in the “Materials and methods” section “BrdU labeling assay” of this paper.

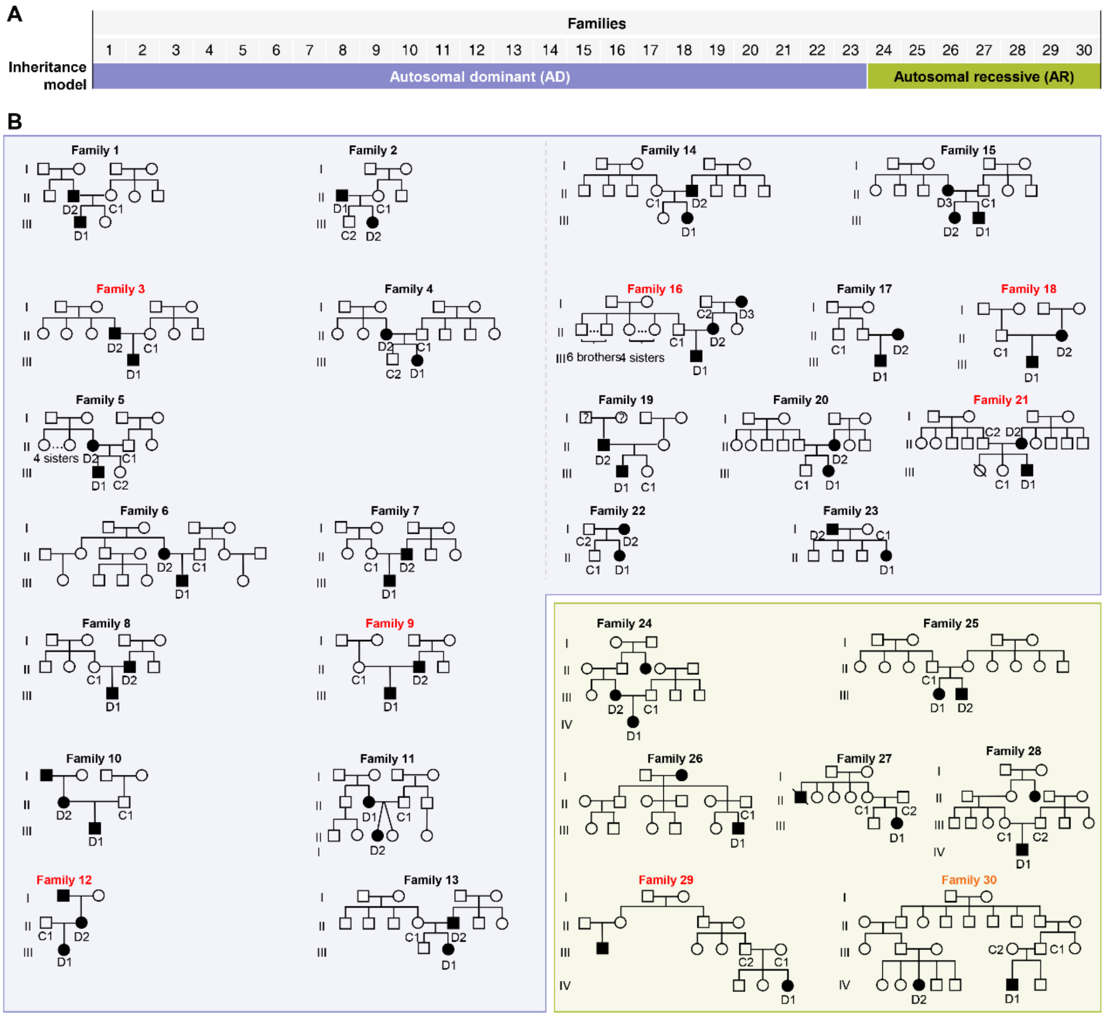

Figure S1. Pedigrees of 30 families with NSOFC.

(A) Among the 30 families with NSOFC, 1-23 families exhibit autosomal dominant (AD) inheritance and 24-30 families exhibit autosomal recessive (AR) inheritance. **(B)** Circles indicate female members while squares male members; black symbols denote NSOFC patients while blank symbols unaffected members. Families with affected member carrying variants in well-known OFC-related pathways are marked in red. Family 30, in which the *ECPAS* variant was identified, is marked in orange.

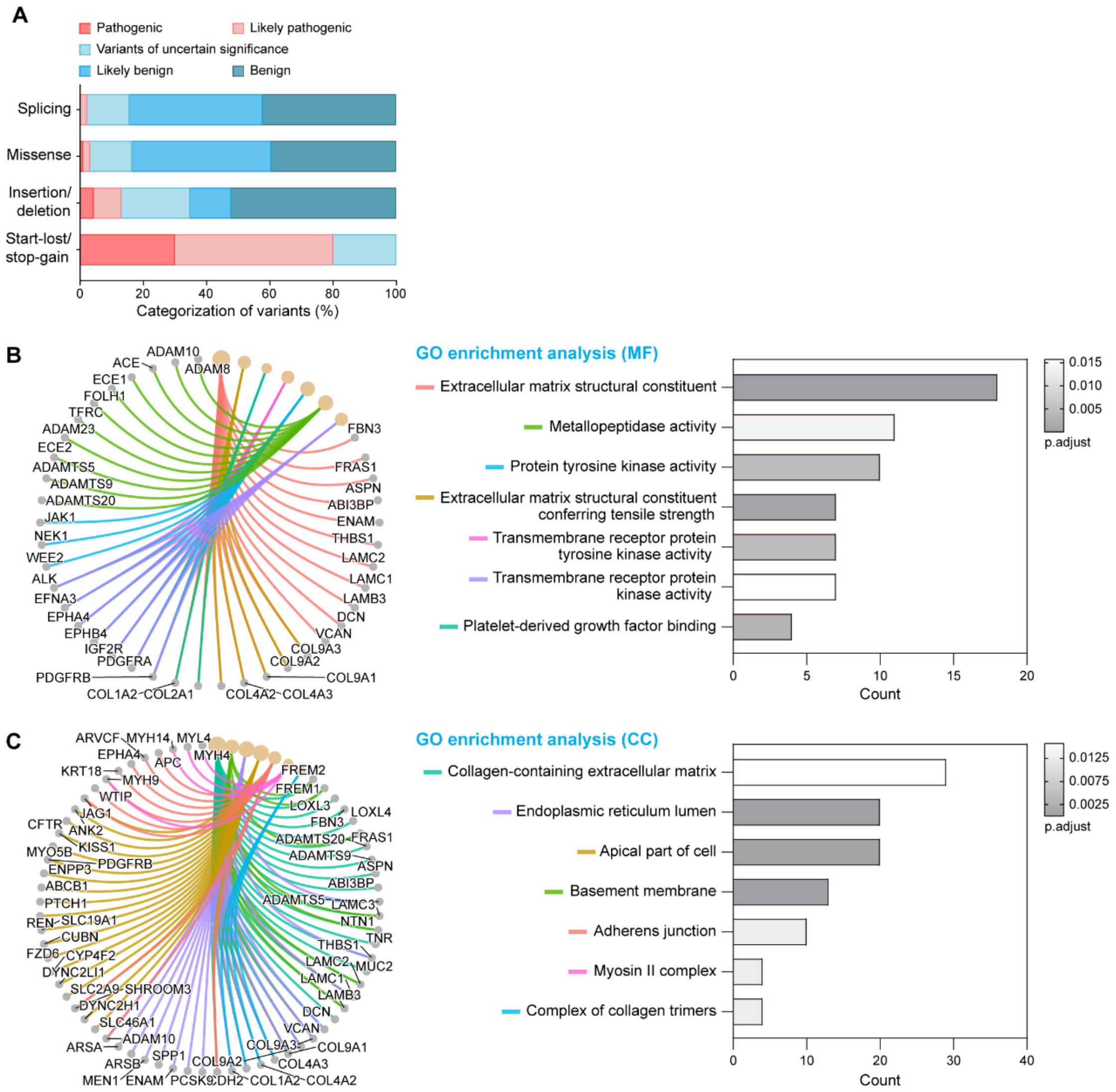

Figure S2. Genetic architecture constructed from 394 candidate variants related to OFC/craniofacial development in these 30 hereditary families.

(A) Categorization of variants according to ACMG guidelines. After applying filtering criteria, we identified a total of 394 candidate variants related to OFC or craniofacial development in these 30 hereditary families, which could be categorized into four types: splicing, missense, insertion/deletion and start-lost/stop-gain. Following the American College of Medical Genetics and Genomics (ACMG) criteria, the majority of start-lost/stop-gain variants were classified as pathogenic or likely pathogenic. Insertion/deletion variants constituted the second highest proportion of pathogenic or likely pathogenic variants and the highest proportion of variants with uncertain significance. In contrast, splicing and missense variants constituted the lowest proportion of pathogenic/likely pathogenic variants. **(B and C)** GO enrichment

analysis of the candidate 394 candidate variants in these 30 hereditary families. To gain further insights into the pool of candidate genes, we performed gene ontology (GO) analysis using clusterProfiler software, which revealed several significant GO terms, including the extracellular matrix structural constituent, collagen-containing extracellular matrix, endoplasmic reticulum lumen, adherens junction, *etc*.

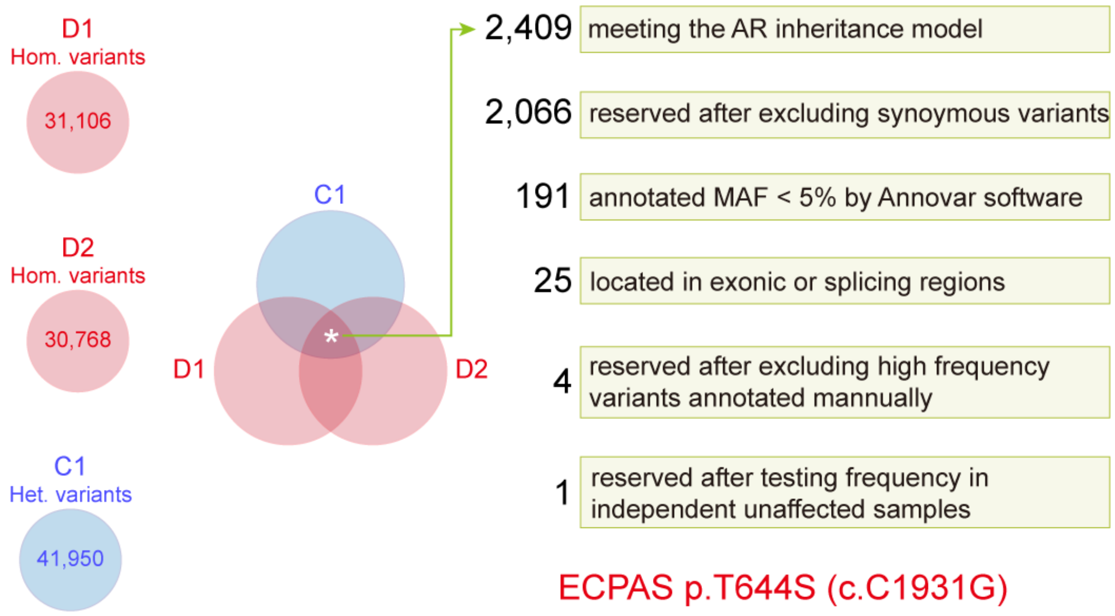

Figure S3. Flowchart illustrating the process of identifying the ECPAS p.T644S (c.C1931G) variant as the candidate variant in Family 30.

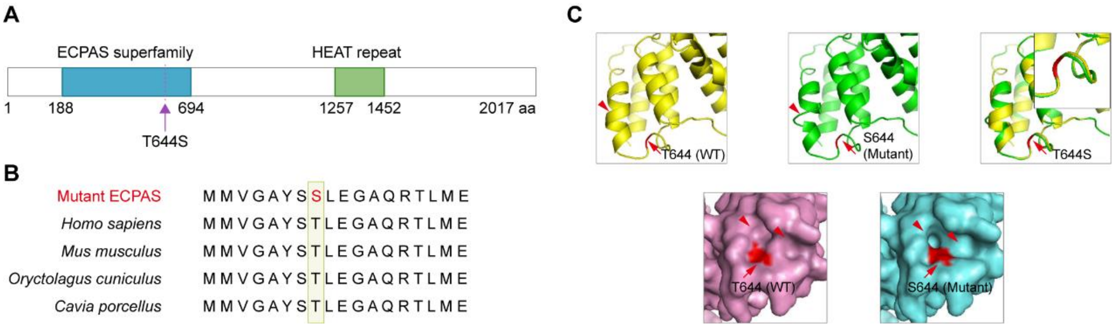

Figure S4. *In silico* prediction of the pathogenicity of T644S variant.

(A) Location of T644S variant in the ECPAS protein. **(B)** Sequence alignment of ECPAS protein from different species revealing the conservation of p.T644. **(C)** Homology predicted models of the wild-type (WT) and T644S mutant (Mutant) ECPAS proteins suggesting that the T644S variant might affect the structure of ECPAS. The upper panel presents a ribbon illustration, while the lower panel depicts the surface electron cloud illustration. Arrows indicate the mutated loci, and arrowheads indicate potentially affected structures.

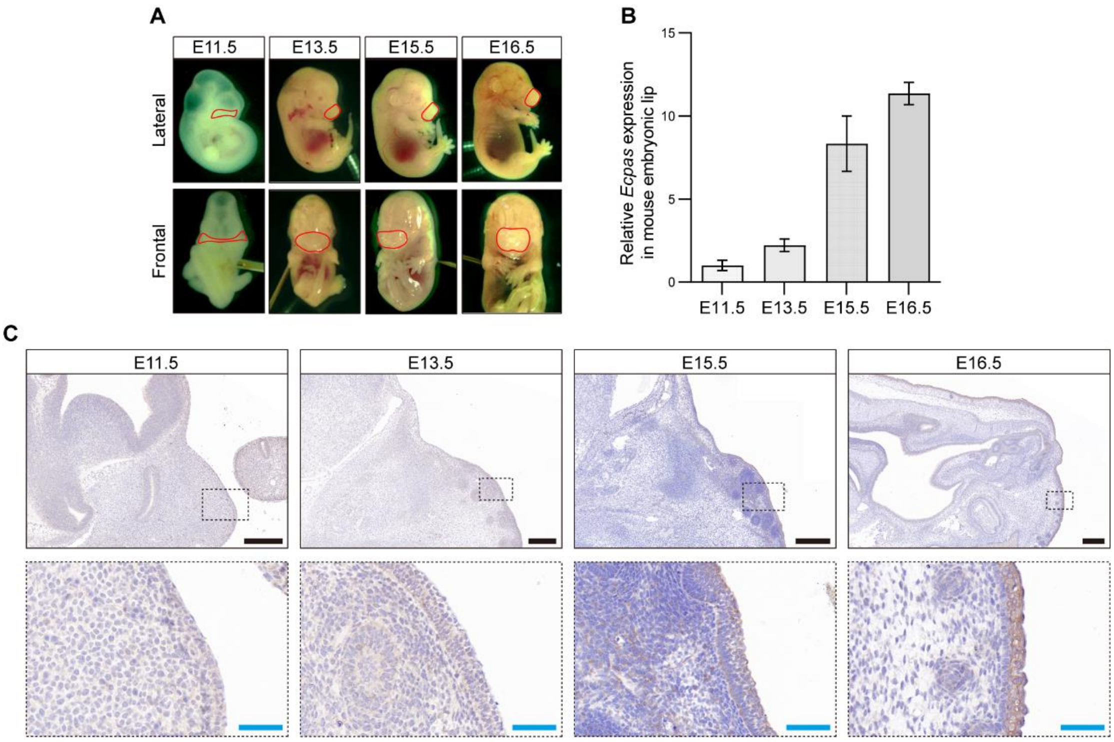

Figure S5. ECPAS expression gradually increases during mouse lip development. **(A)** Lip tissues, circled by red lines, were isolated from mouse embryos at indicated developmental stages. **(B)** The expression of *Ecpas* was measured by qPCR with specific primers (F primer: 5'-GCTCCGACTCAGATCAGCTC-3'; R primer: 5'-CTTCTTGGGTGCTGGACAGT-3'). Data represent mean ± SD. **(C)** Sagittal sections of embryonic upper lips from wild-type mice stained with ECPAS antibody at E11.5, E13.5, E15.5, and E16.5 revealed a steady increase in ECPAS expression within the epithelium of the developing lip. Nucleus was stained with haematoxylin. Black dashed boxes indicating areas magnified for detailed observation. Scale bar (black), 250 μm; scale bar (blue), 50 μm.

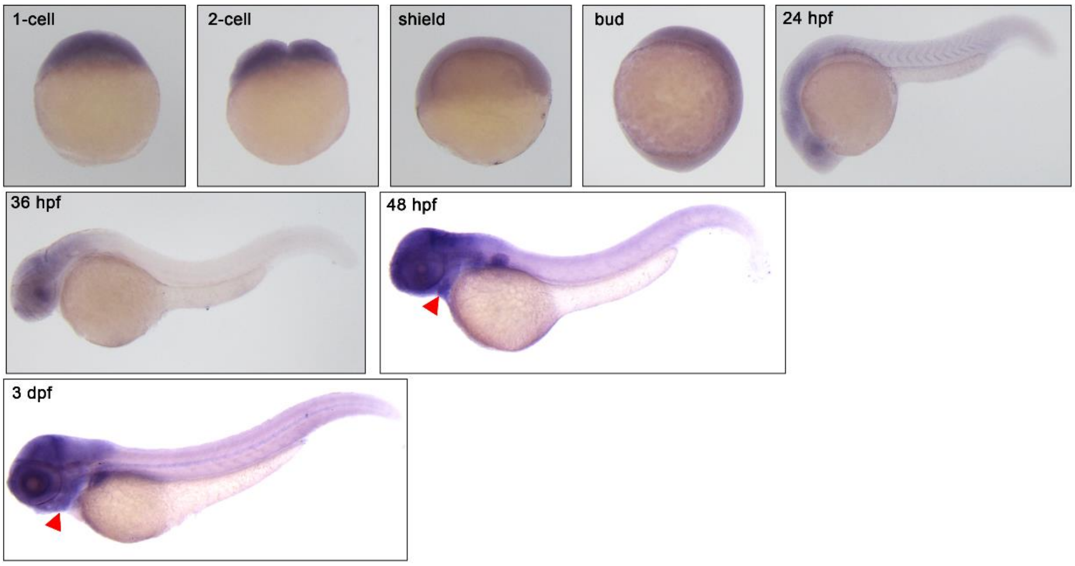

Figure S6. *ecpas* expression pattern in zebrafish embryos.

Expression pattern of *ecpas* at different stages (1-cell stage to 3 dpf) as detected by ISH. Notably, *ecpas* expression was observed in the craniofacial region from 36 hpf to 3 dpf. Red arrowheads indicate the pharyngeal region.

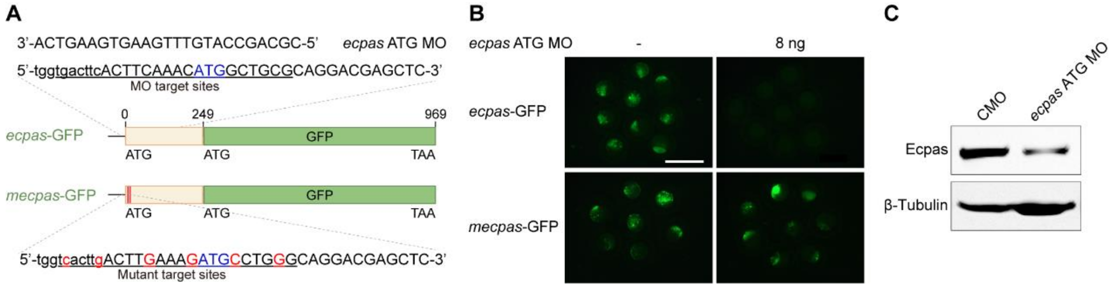

Figure S7. Validation of zebrafish *ecpas* ATG MO.

(A) The sequences of zebrafish *ecpas* translation-blocking morpholino oligonucleotide (MO) and its targeting sites are shown. Note that the several nucleotides in targeting sites are substituted in mutant *ecpas*-GFP (*mecpas*-GFP) construct. **(B)** Zebrafish embryos were injected with negative control or *ecpas* MO along with *ecpas-GFP* or *mecpas-GFP* mRNA as indicated. *ecpas* MO blocked translation of *ecpas-GFP* mRNA, but not mecpas-*GFP* mRNA while negative control MO had no effect. **(C)** Validation of *ecpas* ATG MO efficiency via Western blot analysis.

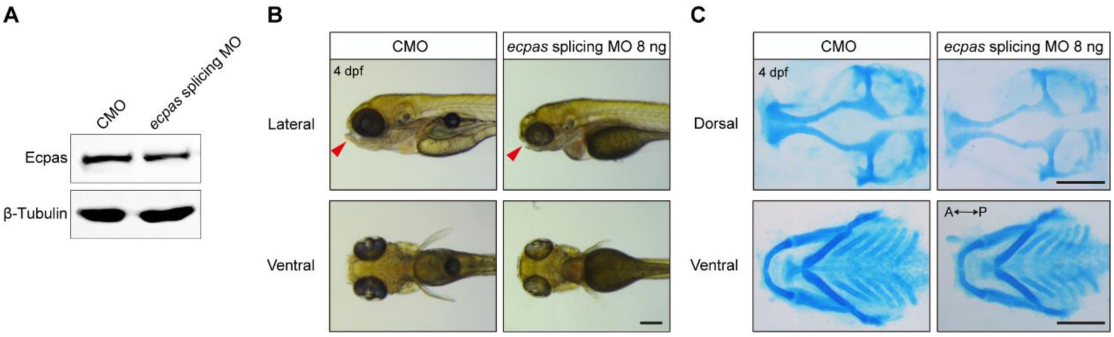

Figure S8. Craniofacial dysplasia induced by Ecpas knockdown via *ecpas* splicing MO.

(A) Validation of *ecpas* splicing MO efficiency via Western blot analysis. **(B)** Lateral and ventral views of zebrafish head morphology at 4 dpf. Microinjection with *ecpas* splicing MO, but not control MO (CMO), resulted in craniofacial dysplasia, characterized by a less protruding mouth. Notably, this phenotype, when compared to that induced by *ecpas* ATG MO, was milder (refer to Figure 2). Red arrowheads indicate the mouth region. Scale bar, 200 µm. **(C)** Dorsal and ventral views of head cartilages, stained with Alcian blue at 4 dpf. Zebrafish embryos microinjected with *ecpas* splicing MO displayed smaller head cartilages, again akin to but less severe than the phenotype induced by *ecpas* ATG MO (refer to Figure 2). Scale bar, 200 µm.

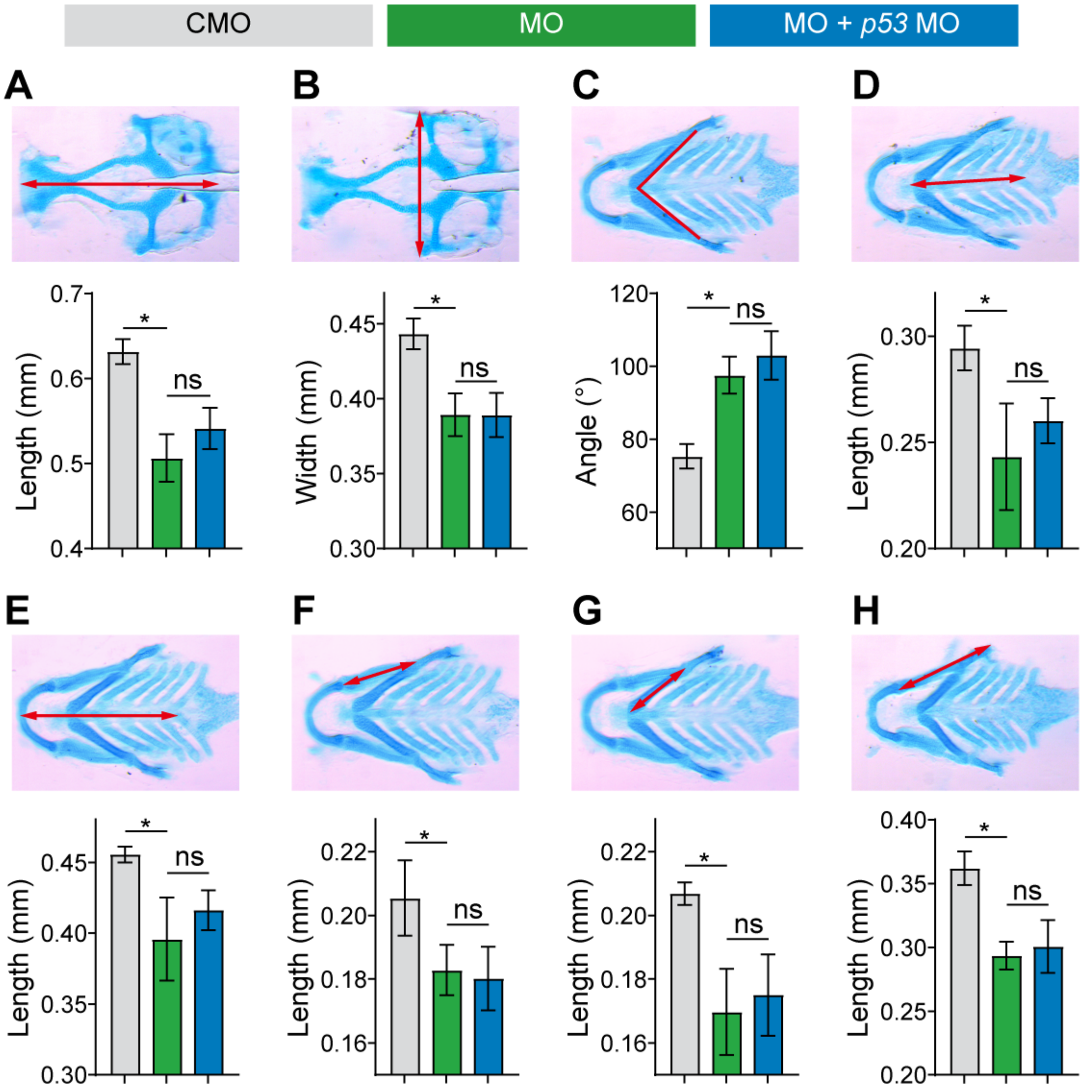

Figure S9. Measurements of craniofacial cartilages following *ecpas* knockdown in zebrafish embryos at 4 dpf (refer to Figure 2B in the main text).

(A and B) Dorsal indicators (n = 3-7 embryos per group): (A) length of the dorsal cartilage and (B) width of the dorsal cartilage. **(C-H)** Ventral indicators (n = 4-6 embryos per group): (C) angle between the ceratohyal cartilages, (D) midline length from the anterior end of the ceratohyal cartilage to the posterior end of the basibranchial bone, (E) midline length from the anterior end of the Meckel's cartilage to the posterior end of the basibranchial bone, (F) length of the palatoquadrate, (G) length of the ceratohyal cartilage and (H) length of the palatoquadrate and hyosymplectic cartilage. Data represent mean ± SD. One-way AVONA for statistical analysis. *, $P < 0.05$; ns, not significant.

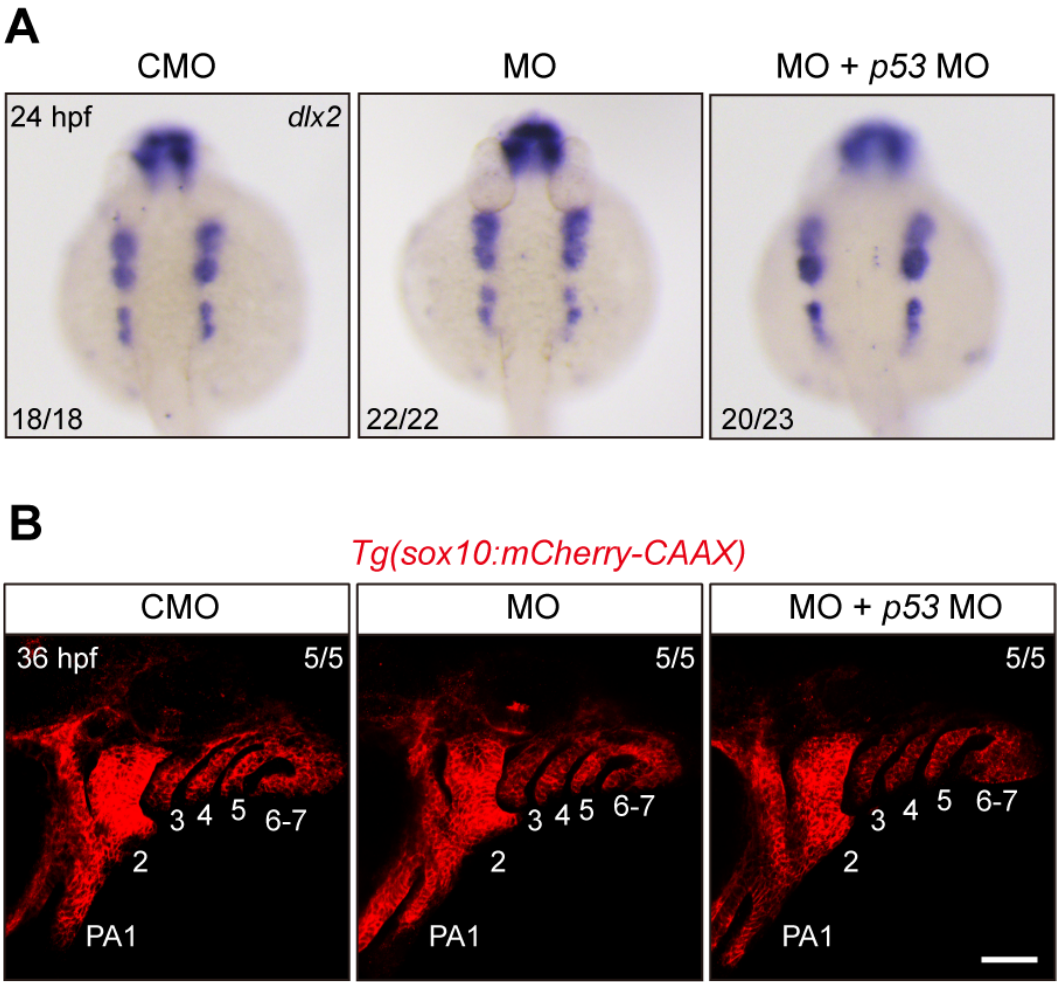

Figure S10. No effect of *ecpas* disruption on both the early and later stage of CNCCs migration was observed.

(A) Whole-mount *in situ* hybridization of *dlx2* (purple) at 24 hpf showing the early migration of CNCCs. **(B)** Lateral views of the later migration of CNCCs (red) at 36 hpf in *Tg(sox10:mCherry-CAAX)* transgenic zebrafish embryos. Scale bar, 50 μm. CNCCs, cranial neural crest cells. CMO, the negative control MO; MO, MO targeting *ecpas* ATG; *p53* MO, MO targeting *p53*. Hpf, hours post-fertilization.

References for the supplemental materials

Cingolani P, Platts A, Wang le L *et al.* A program for annotating and predicting the effects of single nucleotide polymorphisms, SnpEff: SNPs in the genome of Drosophila melanogaster strain w1118; iso-2; iso-3. *Fly (Austin)* 2012;**6**:80-92.

DePristo MA, Banks E, Poplin R *et al.* A framework for variation discovery and genotyping using next-generation DNA sequencing data. *Nat Genet* 2011;**43**:491-498.

Dixon MJ, Marazita ML, Beaty TH *et al.* Cleft lip and palate: understanding genetic and environmental influences. *Nat Rev Genet* 2011;**12**:167-178.

Dutton JR, Antonellis A, Carney TJ *et al.* An evolutionarily conserved intronic region controls the spatiotemporal expression of the transcription factor Sox10. *BMC Dev Biol* 2008;**8**:105.

Huang W, He Q, Li M *et al.* Two rare variants reveal the significance of Grainyhead-like 3 Arginine 391 underlying non-syndromic cleft palate only. *Oral Dis* 2023a;**29**:1632-1643.

Huang W, Zhang S, Lin J *et al.* Rare loss-of-function variants in FLNB cause non-syndromic orofacial clefts. *J Genet Genomics* 2023b.

Kimmel CB, Ballard WW, Kimmel SR *et al.* Stages of embryonic development of the zebrafish. *Dev Dyn* 1995;**203**:253-310.

Kopanos C, Tsiolkas V, Kouris A *et al.* VarSome: the human genomic variant search engine. *Bioinformatics* 2019;**35**:1978-1980.

Lawson ND, and Weinstein BM. In vivo imaging of embryonic vascular development using transgenic zebrafish. *Dev Biol* 2002;**248**:307-318.

Liu J, Zhang Y, Zhang L *et al.* Orofacial Clefts in High Prevalence Area of Birth Defects - Five Counties, Shanxi Province, China, 2000-2020. *China CDC Wkly* 2021;**3**:773-777.

Massenburg BB, Hopper RA, Crowe CS *et al.* Global Burden of Orofacial Clefts and the World Surgical Workforce. *Plast Reconstr Surg* 2021;**148**:568e-580e.

McKusick VA. AUTOSOMAL DOMINANT PHENOTYPES. 1966a;In Mendelian

Inheritance in Man, V.A. McKusick, ed. (Butterworth-Heinemann), pp. 1-137.

McKusick VA. AUTOSOMAL RECESSIVE PHENOTYPES. 1966b;In Mendelian Inheritance in Man, V.A. McKusick, ed. (Butterworth-Heinemann), pp. 139-246.

Meng P, Zhao H, Huang W *et al.* Three GLI2 mutations combined potentially underlie non-syndromic cleft lip with or without cleft palate in a Chinese pedigree. *Mol Genet Genomic Med* 2019;**7**:e714.

Mork L, and Crump G. Zebrafish Craniofacial Development: A Window into Early Patterning. *Curr Top Dev Biol* 2015;**115**:235-269.

Ning G, Liu X, Dai M *et al.* MicroRNA-92a upholds Bmp signaling by targeting noggin3 during pharyngeal cartilage formation. *Dev Cell* 2013;**24**:283-295.

Rezaei M, Basiri M, Hasani SN *et al.* Establishment of a Transgenic Zebrafish Expressing GFP in the Skeletal Muscle as an Ornamental Fish. *Galen Med J* 2019;**8**:e1068.

Richards S, Aziz N, Bale S *et al.* Standards and guidelines for the interpretation of sequence variants: a joint consensus recommendation of the American College of Medical Genetics and Genomics and the Association for Molecular Pathology. *Genet Med* 2015;**17**:405-424.

Rodrigues FS, Doughton G, Yang B *et al.* A novel transgenic line using the Cre-lox system to allow permanent lineage-labeling of the zebrafish neural crest. *Genesis* 2012;**50**:750-757.

Takeuchi M, Kaneko H, Nishikawa K *et al.* Efficient transient rescue of hematopoietic mutant phenotypes in zebrafish using Tol2-mediated transgenesis. *Dev Growth Differ* 2010;**52**:245-250.

Tavtigian SV, Harrison SM, Boucher KM *et al.* Fitting a naturally scaled point system to the ACMG/AMP variant classification guidelines. *Hum Mutat* 2020;**41**:1734-1737.

Vikkula M, Mariman EC, Lui VC *et al.* Autosomal dominant and recessive osteochondrodysplasias associated with the COL11A2 locus. *Cell* 1995;**80**:431-437.

Wang X, Chemmama IE, Yu C *et al.* The proteasome-interacting Ecm29 protein

disassembles the 26S proteasome in response to oxidative stress. *J Biol Chem* 2017;**292**:16310-16320.

Yang H, Robinson PN, and Wang K. Phenolyzer: phenotype-based prioritization of candidate genes for human diseases. *Nat Methods* 2015;**12**:841-843.

Zhang F, Zhu X, Wang P *et al.* The cytokine FAM3B/PANDER is an FGFR ligand that promotes posterior development in Xenopus. *Proc Natl Acad Sci U S A* 2021;**118**.

Zhang J, Zhao H, Huang W *et al.* A novel FZD6 mutation revealed the cause of cleft lip and/or palate in a Chinese family. *Genes & Diseases* 2020;**7**:440-447.

Zhao H, Zhang M, Zhong W *et al.* A novel IRF6 mutation causing non-syndromic cleft lip with or without cleft palate in a pedigree. *Mutagenesis* 2018a;**33**:195-202.

Zhao H, Zhong W, Leng C *et al.* A novel PTCH1 mutation underlies nonsyndromic cleft lip and/or palate in a Han Chinese family. *Oral Dis* 2018b;**24**:1318-1325.

Zhong W, Zhao H, Huang W *et al.* Identification of rare PTCH1 nonsense variant causing orofacial cleft in a Chinese family and an up-to-date genotype-phenotype analysis. *Genes & Diseases* 2020.

Table S1. 394 candidate variants related to OFC or craniofacial development identified in 30 hereditary families after applying filtering criteria.

Family ID	Gene	Zygosity	Genomic position	cDNA change	Protein change	ACMG category
1	*CFTR*	Het	chr7:117149150	c.233dup	p.Trp79Leufs	Pathogenic
1	*ALK*	Het	chr2:29940442	c.787+2T>C	-	Likely pathogenic
1	*KMT2D*	Het	chr12:49435098	c.6455G>T	p.Gly2152Val	Likely benign
1	*PREPL*	Het	chr2:44556131	c.1474C>T	p.Pro492Ser	Uncertain significance
1	*KMT2C*	Het	chr7:151875100	c.7443_7444insTTTTTTTTA	-	Likely benign
1	*GOLGB1*	Het	chr3:121417148	c.2222G>A	p.Ser741Asn	Likely benign
1	*ARID1B*	Het	chr6:157100023	c.1229_1234del	p.Gly410_Gly411del	Likely benign
1	*SCARF2*	Het	chr22:20786125	c.241G>A	p.Glu81Lys	Likely benign
1	*PYGO2*	Het	chr1:154933953	c.100G>T	p.Ala34Ser	Likely benign
1	*ZNF714*	Het	chr19:21300884	c.1414A>T	p.Ile472Leu	Likely benign
1	*COL4A3*	Het	chr2:228125837	c.1150+4T>C	-	Likely benign
1	*SYNJ2*	Het	chr6:158508015	c.3337C>A	p.Pro1113Thr	Likely benign
1	*MUC2*	Het	chr11:1093657	c.5476C>G	p.Pro1826Ala	Likely benign
1	*PWP2*	Het	chr21:45545943	c.2017G>C	p.Asp673His	Benign
2	*FGF5*	Het	chr4:81207591	c.572C>T	p.Ala191Val	Uncertain significance
2	*IBSP*	Het	chr4:88732979	c.871C>T	p.Arg291*	Uncertain significance

2	*KMT2C*	Het	chr7:151878905	c.6040G>A	p.Ala2014Thr	Likely benign
3	*CREBBP*	Het	chr16:3819324	c.2911A>G	p.Arg971Gly	Likely pathogenic
3	*MEN1*	Het	chr11:64572131	c.1523G>A	p.Gly508Asp	Benign
3	*FKBP5*	Het	chr6:35586902	c.479C>T	p.Ser160Leu	Uncertain significance
3	*VCAN*	Het	chr5:82836692	c.7870G>A	p.Glu2624Lys	Likely benign
3	*LOXL4*	Het	chr10:100013406	c.1739G>A	p.Arg580His	Uncertain significance
3	*ZNF189*	Het	chr9:104171711	c.1661G>C	p.Gly554Ala	Likely benign
3	*KRT18*	Het	chr12:53343099	c.142G>A	p.Val48Met	Likely benign
3	*FREM1*	Het	chr9:14792862	c.3860A>G	p.Asn1287Ser	Likely benign
3	*SEMA4A*	Het	chr1:156145400	c.1646G>A	p.Ser549Asn	Likely benign
3	*E2F3*	Het	chr6:20402533	c.70G>A	p.Val24Ile	Likely benign
3	*CR1*	Het	chr1:207753750	c.5102C>T	p.Pro1701Leu	Likely benign
3	*SPECC1L*	Het	chr22:24717843	c.895A>G	p.Thr299Ala	Benign
3	*RUNX3*	Het	chr1:25256278	c.121_123dup	p.Gly41dup	Benign
3	*SLC30A1*	Het	chr1:211748819	c.1435A>C	p.Lys479Gln	Benign
3	*KLF5*	Het	chr13:73636050	c.313A>G	p.Ile105Val	Benign
3	*COL9A2*	Het	chr1:40770042	c.1237C>T	p.Pro413Ser	Benign
4	*KIF7*	Het	chr15:90176972	c.2537C>T	p.Thr846Met	Likely benign
4	*GOLGA2*	Het	chr9:131038251	c.-3_4delCTGATGT	-	Likely pathogenic

4	*TTC28*	Het	chr22:29075651	c.61C>T	p.Arg21*	Likely pathogenic
4	*GOLGA2*	Het	chr9:131038250	c.6G>C	p.Trp2Cys	Likely benign
4	*DOLK*	Het	chr9:131708945	c.638T>C	p.Leu213Pro	Uncertain significance
4	*CD59*	Het	chr11:33731717	c.339_341delTCT	p.Leu115del	Uncertain significance
4	*ATM*	Het	chr11:108192078	c.6503C>T	p.Ser2168Leu	Uncertain significance
4	*FRAS1*	Het	chr4:79372921	c.6464-5T>G	-	Likely benign
4	*LAMC3*	Het	chr9:133944405	c.2858G>A	p.Gly953Asp	Likely benign
4	*MUC5B*	Het	chr11:1268288	c.10178C>T	p.Thr3393Met	Likely benign
4	*TNR*	Het	chr1:175362998	c.1274C>T	p.Thr425Met	Benign
5	*PHLPP1*	Het	chr18:60527731	c.1963A>C	p.Asn655His	Likely benign
5	*BMP5*	Het	chr6:55684638	c.498A>C	p.Arg166Ser	Likely benign
5	*DROSHA*	Het	chr5:31526531	c.509C>T	p.Pro170Leu	Benign
6	*AXIN2*	Het	chr17:63554027	c.712G>A	p.Asp238Asn	Uncertain significance
6	*IL23R*	Het	chr1:67635269	c.315T>G	p.Cys105Trp	Uncertain significance
6	*COL9A1*	Het	chr6:70935688	c.2528G>A	p.Arg843His	Uncertain significance
6	*SYNE1*	Het	chr6:152831457	c.452G>T	p.Ser151Ile	Uncertain significance
6	*PPP3CC*	Het	chr8:22368728	c.614T>C	p.Leu205Ser	Uncertain significance
6	*MLH1*	Het	chr3:37053569	c.656T>C	p.Ile219Thr	Uncertain

						significance
6	*HERC2*	Het	chr15:28478342	c.4625G>A	p.Arg1542His	Likely benign
6	*CHRNG*	Het	chr2:233410418	c.1546C>G	p.Pro516Ala	Likely benign
6	*CRISPLD1*	Het	chr8:75898156	c.-62-5T>C	-	Likely benign
6	*RRM2B*	Het	chr8:103220504	c.1129G>T	p.Ala377Ser	Likely benign
6	*PGAP3*	Het	chr17:37844093	c.175C>G	p.Leu59Val	Likely benign
6	*IBSP*	Het	chr4:88732782	c.674C>T	p.Ser225Leu	Likely benign
6	*HSPA13*	Het	chr21:15750738	c.367-5C>A	-	Likely benign
6	*HSPA12B*	Het	chr20:3732752	c.2000C>T	p.Thr667Ile	Likely benign
6	*GRM4*	Het	chr6:34122936	c.232G>C	p.Glu78Gln	Likely benign
6	*ALPI*	Het	chr2:233323052	c.1117G>A	p.Val373Ile	Likely benign
6	*ABCB1*	Het	chr7:87145883	c.3026T>C	p.Ile1009Thr	Likely benign
6	*SYNJ2*	Het	chr6:158403093	c.100T>C	p.Phe34Leu	Likely benign
6	*MYO5B*	Het	chr18:47363917	c.5108T>C	p.Val1703Ala	Benign
6	*THBS1*	Het	chr15:39876498	c.904-3T>C	-	Benign
6	*PRDM16*	Het	chr1:3329208	c.2447A>G	p.Asn816Ser	Benign
6	*UBR1*	Het	chr15:43317071	c.2695A>G	p.Ile899Val	Benign
6	*SIX5*	Het	chr19:46269262	c.1717G>C	p.Val573Leu	Benign
7	*IFT88*	Het	chr13:21245158	c.2249G>A	p.Arg750Lys	Likely benign

7	*CELSR1*	Het	chr22:46929722	c.3346T>A	p.Ser1116Thr	Uncertain significance
7	*DACH1*	Het	chr13:72440658	c.244_249delGGCGGC	p.Gly82_Gly83del	Uncertain significance
7	*CD59*	Het	chr11:33738993	c.92G>T	p.Cys31Phe	Likely pathogenic
7	*GOLGB1*	Het	chr3:121435949	c.923A>C	p.His308Pro	Likely benign
7	*COL2A1*	Het	chr12:48379503	c.1680+8G>T	-	Likely benign
7	*LOXHD1*	Het	chr18:44146330	c.2327G>A	p.Arg776His	Likely benign
7	*MYH9*	Het	chr22:36681311	c.5339G>A	p.Arg1780Gln	Likely benign
7	*FOLH1*	Het	chr11:49229868	c.94T>G	p.Phe32Val	Likely benign
7	*PDGFRB*	Het	chr5:149515447	c.41-6C>T	-	Likely benign
7	*TG*	Het	chr8:133918944	c.3646C>G	p.Pro1216Ala	Likely benign
7	*SAMD8*	Het	chr10:76936374	c.1172A>G	p.Asn391Ser	Likely benign
7	*SYNE1*	Het	chr6:152722394	c.6908C>T	p.Thr2303Met	Likely benign
7	*MYH14*	Het	chr19:50750345	c.1295G>A	p.Arg432Gln	Likely benign
7	*KCNH4*	Het	chr17:40330149	c.554G>A	p.Arg185His	Likely benign
7	*JAK1*	Het	chr1:65304196	c.2919C>A	p.Asn973Lys	Likely benign
7	*CYFIP1*	Het	chr15:22962463	c.2183G>A	p.Arg728Gln	Likely benign
7	*CHDH*	Het	chr3:53851962	c.1627G>A	p.Val543Ile	Likely benign
7	*SHROOM3*	Het	chr4:77660063	c.737T>C	p.Ile246Thr	Benign
7	*ACACB*	Het	chr12:109613826	c.1307A>G	p.Asp436Gly	Benign

7	*KCNH4*	Het	chr17:40312080	c.3032C>A	p.Ser1011Tyr	Benign
8	*RPS19*	Het	chr19:42365237	c.128A>G	p.Lys43Arg	Uncertain significance
8	*LAMB3*	Het	chr1:209807877	c.479T>G	p.Val160Gly	Uncertain significance
8	*ADAM10*	Het	chr15:58891920	c.2029C>T	p.His677Tyr	Uncertain significance
8	*ADAMTS9*	Het	chr3:64641303	c.1118C>T	p.Ser373Leu	Likely benign
8	*RYK*	Het	chr3:133921659	c.701G>T	p.Gly234Val	Likely benign
8	*TERT*	Het	chr5:1293685	c.1316A>G	p.Glu439Gly	Likely benign
8	*ERCC6*	Het	chr10:50667028	c.4315G>C	p.Ala1439Pro	Likely benign
8	*SYNE1*	Het	chr6:152603091	c.18232G>A	p.Glu6078Lys	Likely benign
8	*GABBR2*	Het	chr9:101148060	c.1530-6T>C	-	Benign
8	*NFATC1*	Het	chr18:77246317	c.2162G>A	p.Gly721Glu	Benign
8	*GABRG3*	Het	chr15:27772767	c.1054A>G	p.Thr352Ala	Benign
8	*CELSR1*	Het	chr22:46759942	c.8986G>A	p.Ala2996Thr	Benign
8	*TACR3*	Het	chr4:104577382	c.857A>G	p.Lys286Arg	Benign
8	*ECE1*	Het	chr1:21551823	c.1960G>A	p.Val654Met	Benign
8	*ASXL2*	Het	chr2:25965359	c.3847C>T	p.Arg1283Cys	Benign
9	*PLEKHA5*	Het	chr12:19406939	c.293T>C	p.Ile98Thr	Likely pathogenic
9	*DHCR7*	Het	chr11:71146584	1253_1264dup	p.Tyr418_Ala421dup	Uncertain significance

9	*RYR1*	Het	chr19:39061316	c.13729A>G	p.Ile4577Val	Uncertain significance
9	*FREM1*	Het	chr9:14775981	c.4663C>G	p.Leu1555Val	Uncertain significance
9	*RHPN2*	Het	chr19:33490487	c.1225+5G>A	-	Uncertain significance
9	*DHX34*	Het	chr19:47861136	c.1031C>T	p.Pro344Leu	Uncertain significance
9	*SYNE1*	Het	chr6:152466619	c.24977-1719A>G	-	Uncertain significance
9	*TNS1*	Het	chr2:218682830	c.3913G>A	p.Gly1305Arg	Likely benign
9	*FAM20C*	Het	chr7:286468	c.952_953insGACAGGTGAGCCCTTCCTTCCTCCCTCCATCCGC	p.Asp318_Arg319fs	Likely pathogenic
9	*CYP1A1*	Het	chr15:75012894	c.1475C>G	p.Pro492Arg	Likely benign
9	*SEPT9*	Het	chr17:75398198	c.134G>A	p.Arg45Gln	Likely benign
9	*ZNF528*	Het	chr19:52919388	c.1283G>A	p.Arg428Gln	Likely benign
9	*ACE*	Het	chr17:61562680	c.2005G>A	p.Glu669Lys	Likely benign
9	*HS6ST1*	Het	chr2:129025795	c.1177G>A	p.Asp393Asn	Benign
9	*ERCC8*	Het	chr5:60194107	c.839C>A	p.Thr280Lys	Benign
10	*SLC25A13*	Het	chr7:95751279	c.1625C>A	p.Ala542Asp	Likely pathogenic
10	*SIM2*	Het	chr21:38081480	c.188C>T	p.Ala63Val	Benign
10	*PDS5A*	Het	chr4:39915285	c.938A>G	p.Lys313Arg	Uncertain significance
10	*RHPN2*	Het	chr19:33490487	c.1225+5G>A	-	Uncertain significance

10	*FBXO11*	Het	chr2:48066644	c.361-5T>C	-	Benign
10	*TSHZ1*	Het	chr18:72997838	c.341C>G	p.Thr114Arg	Likely benign
10	*RYR1*	Het	chr19:38934191	c.271-7C>G	-	Likely benign
10	*ASXL1*	Het	chr20:31022647	c.2132C>T	p.Thr711Ile	Likely benign
10	*EPB41L2*	Het	chr6:131199237	c.2043+7C>T	-	Likely benign
10	*DCN*	Het	chr12:91539959	c.956C>T	p.Thr319Ile	Likely benign
10	*ARVCF*	Het	chr22:19978229	c.89T>C	p.Leu30Pro	Likely benign
10	*ZNF528*	Het	chr19:52909255	c.111G>T	p.Met37Ile	Likely benign
10	*DYNC2H1*	Het	chr11:103004281	c.1954-3T>C	-	Likely benign
10	*ADCY9*	Het	chr16:4016924	c.2914C>T	p.Arg972Trp	Benign
10	*RAD54B*	Het	chr8:95479680	c.88C>G	p.Leu30Val	Benign
10	*SLC6A4*	Het	chr17:28548904	c.73G>A	p.Gly25Arg	Benign
10	*LAMC1*	Het	chr1:183097889	c.3280+4C>T	-	Benign
10	*HOXB2*	Het	chr17:46620792	c.709G>A	p.Ala237Thr	Benign
10	*ABR*	Het	chr17:934893	c.89C>G	p.Pro30Arg	Benign
11	*NOTCH3*	Het	chr19:15288391	c.4348G>A	p.Ala1450Thr	Benign
11	*ABCA4*	Het	chr1:94508434	c.3211T>A	p.Ser1071Thr	Pathogenic
11	*LPL*	Het	chr8:19809418	c.388C>A	p.Leu130Met	Uncertain significance
11	*ADAMTS20*	Het	chr12:43825284	c.3112G>A	p.Gly1038Ser	Uncertain significance

11	*DCAF4L2*	Het	chr8:88886001	c.199T>C	p.Ser67Pro	Likely benign
11	*NAPA*	Het	chr19:48018133	c.65A>G	p.Lys22Arg	Likely benign
11	*BAX*	Het	chr19:49458217	c.32G>A	p.Gly11Glu	Likely benign
11	*COL9A3*	Het	chr20:61467883	c.1602C>T	-	Likely benign
11	*MN1*	Het	chr22:28195186	c.1346C>G	p.Pro449Arg	Benign
11	*ZEB2*	Het	chr2:145274839	c.73+6A>C	-	Benign
11	*IGF2R*	Het	chr6:160469523	c.2462C>T	p.Pro821Leu	Benign
11	*NEDD4L*	Het	chr18:56008406	c.1257+5G>A	-	Benign
11	*MUC5B*	Het	chr11:1254237	c.2066-6C>T	-	Benign
12	*PTCH1*	Het	chr9:98241322	c.1175C>T	p.Ala392Val	Pathogenic
12	*TBX3*	Het	chr12:115112328	c.1412G>A	p.Arg471His	Uncertain significance
12	*COL9A2*	Het	chr1:40775653	c.803G>A	p.Gly268Asp	Uncertain significance
12	*ARID1B*	Het	chr6:157527461	c.5186G>A	p.Gly1729Glu	Likely benign
12	*MN1*	Het	chr22:28195216	c.1316A>G	p.Asn439Ser	Likely benign
12	*KRT18*	Het	chr12:53343105	c.148C>T	p.Arg50Cys	Likely benign
12	*SEMA3D*	Het	chr7:84628858	c.2232C>A	p.Asn744Lys	Likely benign
12	*LEFTY1*	Het	chr1:226075549	c.428_433delCCCGGG	p.Ala143_Arg144del	Likely benign
12	*HOXA1*	Het	chr7:27135310	c.216_221delTCGCCA	p.Arg73_His74del	Uncertain significance
12	*ARSA*	Het	chr22:51063695	c.1408G>A	p.Ala470Thr	Likely benign

12	*BLM*	Het	chr15:91292676	c.178T>A	p.Leu60Ile	Likely benign
12	*NEUROG2*	Het	chr4:113436420	c.212C>T	p.Ala71Val	Likely benign
12	*DTNB*	Het	chr2:25611154	c.1652C>T	p.Thr551Met	Likely benign
12	*KMT2D*	Het	chr12:49427347	c.11141G>A	p.Arg3714Lys	Benign
12	*CDH2*	Het	chr18:25593770	c.276C>G	p.Ser92Arg	Benign
12	*PPM1D*	Het	chr17:58733979	c.1037G>T	p.Cys346Phe	Benign
12	*MUC5B*	Het	chr11:1256409	c.2725G>A	p.Asp909Asn	Benign
12	*CR1*	Het	chr1:207782707	c.5969A>G	p.Asn1990Ser	Benign
13	*CREBBP*	Het	chr16:3777792	c.7256C>T	p.Ala2419Val	Likely benign
13	*RECQL4*	Het	chr8:145737671	c.3090_3091delCA	p.Phe1030fs	Likely pathogenic
13	*KMT2D*	Het	chr12:49425724	c.12764G>A	p.Gly4255Asp	Uncertain Significance
13	*EPHA4*	Het	chr2:222301246	c.2219T>C	p.Met740Thr	Uncertain significance
13	*TIMELESS*	Het	chr12:56812137	c.3235T>C	p.Phe1079Leu	Uncertain significance
13	*CUBN*	Het	chr10:17146534	c.1301A>G	p.Asn434Ser	Uncertain significance
13	*THADA*	Het	chr2:43801878	c.1326G>T	p.Glu442Asp	Likely benign
13	*PLCB4*	Het	chr20:9319533	c.226-8C>T	-	Likely benign
13	*C5orf42*	Het	chr5:37176029	c.5960C>G	p.Thr1987Arg	Likely benign
13	*SALL3*	Het	chr18:76757228	c.3809C>T	p.Pro1270Leu	Likely benign

13	*LOXL3*	Het	chr2:74763574	c.937G>A	p.Ala313Thr	Likely benign
13	*N4BP2*	Het	chr4:40122958	c.3227C>T	p.Thr1076Met	Likely benign
13	*XRCC1*	Het	chr19:44056187	c.1064G>A	p.Arg355Gln	Likely benign
14	*FAT4*	Het	chr4:126242262	c.4696G>A	p.Glu1566Lys	Uncertain significance
14	*PIGN*	Het	chr18:59815450	c.671C>T	p.Ser224Leu	Uncertain significance
14	*MTOR*	Het	chr1:11190768	c.5431C>T	p.Arg1811Cys	Uncertain significance
14	*ADAM8*	Het	chr10:135083903	c.1746T>A	p.Tyr582*	Uncertain significance
14	*PDGFRA*	Het	chr4:55140770	c.1631T>C	p.Val544Ala	Likely benign
14	*REN*	Het	chr1:204135327	c.95A>C	p.Lys32Thr	Likely benign
14	*TULP4*	Het	chr6:158735298	c.250G>A	p.Glu84Lys	Likely benign
14	*SAMD3*	Het	chr6:130530725	c.370G>T	p.Ala124Ser	Likely benign
14	*NEK1*	Het	chr4:170502002	c.859C>G	p.Pro287Ala	Likely benign
14	*KIF27*	Het	chr9:86452373	c.3749A>C	p.Lys1250Thr	Likely benign
14	*BLM*	Het	chr15:91292676	c.178T>A	p.Leu60Ile	Likely benign
14	*SYNE1*	Het	chr6:152674390	c.11253+8C>T	-	Likely benign
14	*PKD2*	Het	chr4:88964613	c.1319+4T>A	-	Likely benign
14	*DYNC2H1*	Het	chr11:103128425	c.10571G>A	p.Arg3524His	Likely benign
14	*EIF4A3*	Het	chr17:78111178	c.983+7C>G	-	Benign
14	*TRAP1*	Het	chr16:3736085	c.383G>A	p.Arg128His	Benign

14	*DHX34*	Het	chr19:47856336	c.49C>T	p.Arg17Trp	Benign
14	*MUC5B*	Het	chr11:1269193	c.11083A>G	p.Lys3695Glu	Benign
15	*SIX3*	Het	chr2:45171742	c.842G>C	p.Arg281Pro	Uncertain significance
15	*PIEZO2*	Het	chr18:10697797	c.6437T>C	p.Met2146Thr	Uncertain significance
15	*LGR5*	Het	chr12:71955587	c.812C>T	p.Ser271Leu	Likely benign
15	*ADAM8*	Het	chr10:135076720	c.2415G>C	p.Lys805Asn	Likely benign
15	*CDKN1B*	Het	chr12:12870998	c.225G>C	p.Glu75Asp	Benign
16	*PTCH1*	Het	chr9:98221936	c.2833C>T	p.Arg945*	Pathogenic
16	*PIEZO2*	Het	chr18:10689681	c.7130T>C	p.Ile2377Thr	Uncertain significance
16	*C5orf42*	Het	chr5:37198917	c.3559G>A	p.Val1187Ile	Likely benign
16	*BARX1*	Het	chr9:96717295	c.134C>A	p.Ala45Glu	Likely benign
16	*BARX1*	Het	chr9:96717296	c.133G>A	p.Ala45Thr	Likely benign
16	*SMG9*	Het	chr19:44251898	c.377C>T	p.Pro126Leu	Likely benign
16	*ALPI*	Het	chr2:233323752	c.1483C>G	p.Leu495Val	Likely benign
16	*CDH4*	Het	chr20:60509244	c.2510C>T	p.Pro837Leu	Benign
16	*SIX5*	Het	chr19:46269262	c.1717G>C	p.Val573Leu	Benign
16	*SIX5*	Het	chr19:46270085	c.1132A>C	p.Ser378Arg	Benign
17	*BHMT*	Het	chr5:78422010	c.767G>A	p.Cys256Tyr	Likely benign
17	*C5orf42*	Het	chr5:37169519	c.6607C>A	p.Pro2203Thr	Likely benign

17	*RNF34*	Het	chr12:121858404	c.754G>A	p.Val252Met	Likely benign
17	*MX2*	Het	chr21:42754475	c.716G>A	p.Arg239Gln	Likely benign
17	*ALK*	Het	chr2:29541241	c.1576G>A	p.Val526Ile	Likely benign
17	*SYNJ2*	Het	chr6:158517193	c.4288C>T	p.Leu1430Phe	Likely benign
17	*PTPRD*	Het	chr9:8492976	c.2353A>G	p.Met785Val	Likely benign
17	*ARHGAP11A*	Het	chr15:32929216	c.2242T>G	p.Leu748Val	Likely benign
17	*MYL4*	Het	chr17:45297318	c.212T>G	p.Met71Arg	Benign
18	*IRF6*	Het	chr1:209974733	c.26G>A	p.Arg9Gln	Pathogenic
18	*ARHGAP29*	Het	chr1:94667305	c.1252G>A	p.Val418Ile	Benign
18	*JAG1*	Het	chr20:10639284	c.526G>A	p.Val176Ile	Benign
18	*LAMC2*	Het	chr1:183155488	c.1A>G	p.Met1?	Pathogenic
18	*SOX6*	Het	chr11:16071472	c.1264G>T	p.Ala422Ser	Likely benign
18	*ERCC6*	Het	chr10:50691463	c.1921C>T	p.His641Tyr	Uncertain significance
18	*PROP1*	Het	chr5:177421267	c.182G>A	p.Gly61Glu	Likely benign
18	*MYO5B*	Het	chr18:47563212	c.455+8T>C	-	Likely benign
18	*PCNT*	Het	chr21:47809221	c.3715C>T	p.Arg1239Cys	Likely benign
18	*KRT18*	Het	chr12:53343007	c.50G>A	p.Gly17Asp	Likely benign
18	*SOX1*	Het	chr13:112722674	c.712_723dup	p.Ala238_His241dup	Likely benign
18	*IL10*	Het	chr1:206944259	c.371G>A	p.Arg124Gln	Likely benign

18	*HSPA12B*	Het	chr20:3730654	c.1081G>C	p.Glu361Gln	Likely benign
18	*EPHB4*	Het	chr7:100417478	c.998G>A	p.Arg333His	Likely benign
18	*DISP1*	Het	chr1:223178995	c.4256A>G	p.Asn1419Ser	Likely benign
18	*WDR11*	Het	chr10:122618231	c.275A>G	p.Asn92Ser	Likely benign
18	*SYNE1*	Het	chr6:152542080	c.21758C>T	p.Ser7253Leu	Likely benign
18	*PTPRD*	Het	chr9:8331584	c.5532C>T	-	Likely benign
18	*LAMB3*	Het	chr1:209807880	c.476G>T	p.Arg159Leu	Likely benign
18	*HERC2*	Het	chr15:28518136	c.815C>T	p.Thr272Met	Likely benign
18	*CYFIP1*	Het	chr15:23002981	c.3703C>T	p.Pro1235Ser	Likely benign
18	*CYFIP1*	Het	chr15:22929903	c.569+8C>T	-	Likely benign
18	*COL4A2*	Het	chr13:111164432	c.5033C>T	p.Thr1678Ile	Benign
19	*BLM*	Het	chr15:91328232	c.2744C>T	p.Ala915Val	Uncertain significance
19	*C2CD3*	Het	chr11:73801894	c.3605A>G	p.Gln1202Arg	Likely benign
19	*SYNE1*	Het	chr6:152740711	c.5414G>A	p.Arg1805Gln	Likely benign
19	*PCSK9*	Het	chr1:55505552	c.63_65dup	p.Leu23dup	Benign
19	*DRD4*	Het	chr11:639550	c.398+12_398+20del	-	Benign
19	*NFATC1*	Het	chr18:77170424	c.149C>T	p.Ser50Phe	Benign
19	*LFNG*	Het	chr7:2552881	c.139_140insGATG	p.Asp47_Gly48fs	Likely pathogenic
20	*FZD2*	Het	chr17:42635585	c.529G>C	p.Gly177Arg	Likely benign

20	*SLC2A9*	Het	chr4:9922067	c.944G>A	p.Trp315*	Pathogenic
20	*POU3F4*	Het	chrX:82763930	c.598C>G	p.Gln200Glu	Uncertain significance
20	*TFRC*	Het	chr3:195778910	c.2186C>T	p.Thr729Met	Likely benign
20	*SLC46A1*	Het	chr17:26731809	c.906A>C	p.Lys302Asn	Likely benign
20	*KRT18*	Het	chr12:53343099	c.142G>A	p.Val48Met	Likely benign
20	*TRIM37*	Het	chr17:57076799	c.2834A>G	p.Asp945Gly	Likely benign
20	*PTPRS*	Het	chr19:5219952	c.3763C>G	p.Pro1255Ala	Benign
20	*CCM2*	Het	chr7:45108137	c.631G>A	p.Val211Met	Benign
20	*PRODH*	Het	chr22:18905991	c.1265A>G	p.Asn422Ser	Benign
21	*GLI2*	Het	chr2:121748112	c.4622C>A	p.Ser1541Tyr	Likely pathogenic
21	*GLI2*	Het	chr2:121747840	c.4350G>T	p.Gln1450His	Likely pathogenic
21	*GLI2*	Het	chr2:121746174	c.2684C>T	p.Ala895Val	Likely pathogenic
21	*C5orf42*	Het	chr5:37206291	c.3149+8T>A	-	Likely benign
21	*SLC19A1*	Het	chr21:46957813	c.61G>A	p.Glu21Lys	Likely benign
21	*TCEB3*	Het	chr1:24077469	c.452G>A	p.Ser151Asn	Likely benign
21	*WDR1*	Het	chr4:10079378	c.1568C>T	p.Ser523Leu	Likely benign
21	*N4BP2*	Het	chr4:40122958	c.3227C>T	p.Thr1076Met	Likely benign
21	*EMX1*	Het	chr2:73145244	c.263C>T	p.Pro88Leu	Likely benign
21	*ANK2*	Het	chr4:114277452	c.7678C>G	p.Pro2560Ala	Likely benign

21	*ACE*	Het	chr17:61562680	c.2005G>A	p.Glu669Lys	Likely benign
21	*ITGB3*	Het	chr17:45361953	c.506G>A	p.Arg169Gln	Benign
21	*KISS1*	Het	chr1:204159922	c.107A>G	p.Gln36Arg	Benign
21	*COL9A2*	Het	chr1:40768332	c.1753G>T	p.Val585Leu	Benign
22	*EVC*	Het	chr4:5754579	c.1115C>T	p.Thr372Met	Likely benign
22	*TTC28*	Het	chr22:29075651	c.61C>T	p.Arg21*	Likely pathogenic
22	*FREM2*	Het	chr13:39424294	c.6499C>T	p.Arg2167Trp	Uncertain significance
22	*VIM*	Het	chr10:17271971	c.550C>G	p.Arg184Gly	Uncertain significance
22	*RBM10*	Het	chrX:47006777	c.92C>G	p.Pro31Arg	Likely benign
22	*NOSIP*	Het	chr19:50063295	c.72G>T	-	Likely benign
22	*UBR1*	Het	chr15:43256191	c.4642A>G	p.Thr1548Ala	Likely benign
22	*STK32B*	Het	chr4:5500734	c.1169A>T	p.Gln390Leu	Likely benign
22	*SPP1*	Het	chr4:88902704	c.333C>A	p.Asp111Glu	Likely benign
22	*MYH4*	Het	chr17:10357965	c.2598G>C	p.Glu866Asp	Likely benign
22	*ACE*	Het	chr17:61561331	c.1708C>T	p.Arg570Trp	Likely benign
22	*DHCR7*	Het	chr11:71155265	c.99-4G>A	-	Benign
22	*FREM1*	Het	chr9:14824088	c.2104A>G	p.Met702Val	Benign
22	*TANC2*	Het	chr17:61497706	c.4363A>G	p.Ile1455Val	Benign
22	*SEMA3A*	Het	chr7:83634712	c.1303G>A	p.Val435Ile	Benign

22	*HDAC4*	Het	chr2:240036742	c.1776+7G>A	-	Benign
22	*CHSY1*	Het	chr15:101718239	c.1763G>C	p.Arg588Thr	Benign
22	*MUC2*	Het	chr11:1094761	c.5837C>A	p.Thr1946Asn	Benign
22	*CHRNA4*	Het	chr20:61981411	c.1352C>T	p.Pro451Leu	Benign
23	*KIF7*	Het	chr15:90177008	c.2501A>G	p.Gln834Arg	Benign
23	*COL1A2*	Het	chr7:94058575	c.3787T>C	p.Tyr1263His	Uncertain significance
23	*COL1A2*	Het	chr7:94039566	c.1048C>T	p.Pro350Ser	Uncertain significance
23	*CASP8*	Het	chr2:202149589	c.1030G>C	p.Asp344His	Likely benign
23	*APC*	Het	chr5:112128186	c.689G>A	p.Arg230His	Likely benign
23	*GLB1L2*	Het	chr11:134226264	c.628G>A	p.Ala210Thr	Likely benign
23	*HERC2*	Het	chr15:28473398	c.5430G>T	p.Met1810Ile	Likely benign
23	*NTN1*	Het	chr17:9124539	c.1466A>G	p.Lys489Arg	Benign
23	*LOXHD1*	Het	chr18:44143153	c.2473G>A	p.Val825Met	Benign
23	*ARSB*	Het	chr5:78135241	c.1151G>A	p.Ser384Asn	Benign
23	*RECQL4*	Het	chr8:145741142	c.1258+6A>T	-	Benign
23	*TNS1*	Het	chr2:218762673	c.16A>G	p.Thr6Ala	Benign
23	*TNS1*	Het	chr2:218683270	c.3473C>T	p.Pro1158Leu	Benign
23	*N4BP2*	Het	chr4:40122313	c.2582A>T	p.Glu861Val	Benign
23	*ZNF541*	Het	chr19:48032829	c.3538+5C>T	-	Benign

23	*IL12RB1*	Het	chr19:18188408	c.587G>A	p.Arg196His	Benign
23	*DMXL1*	Het	chr5:118480316	c.2552G>A	p.Ser851Asn	Benign
23	*CELSR1*	Het	chr22:46763757	c.7953-5C>T	-	Benign
23	*CELSR1*	Het	chr22:46787697	c.5981T>C	p.Leu1994Pro	Benign
23	*CELSR1*	Het	chr22:46760086	c.8842G>A	p.Gly2948Ser	Benign
23	*CELSR1*	Het	chr22:46761497	c.8390G>C	p.Cys2797Ser	Benign
23	*ALDH1L1*	Het	chr3:125873491	c.661-5C>T	-	Benign
23	*ADAMTS5*	Het	chr21:28338298	c.413G>C	p.Gly138Ala	Benign
23	*SEPT9*	Het	chr17:75488774	c.1452C>G	p.Asp484Glu	Benign
23	*SYNE1*	Het	chr6:152776572	c.2881C>T	p.Arg961Trp	Benign
23	*SUMO3*	Het	chr21:46233866	c.175A>G	p.Ser59Gly	Benign
23	*MUC5B*	Het	chr11:1264292	c.6182C>G	p.Ala2061Gly	Benign
23	*LPL*	Het	chr8:19805708	c.106G>A	p.Asp36Asn	Benign
23	*CYP4F2*	Het	chr19:16001215	c.554G>T	p.Gly185Val	Benign
23	*CR1*	Het	chr1:207782707	c.5969A>G	p.Asn1990Ser	Benign
23	*ADAM8*	Het	chr10:135086766	c.565C>T	p.Arg189Trp	Benign
23	*ADAM23*	Het	chr2:207310135	c.319A>G	p.Met107Val	Benign
24	*DCAF8L2*	Hom	chrX:27765399	c.432_434del	p.Glu147del	Benign
24	*HEG1*	Hom	chr3:124732419	c.1986_2003dup	p.Ser667_Ser672dup	Benign

25	*C11orf80*	Hom	chr11:66512290	c.101_103dup	p.Ala34dup	Benign
25	*C6orf223*	Hom	chr6:43970503	c.370_371insGCG	p.Ala124_Ala125insAla	Benign
25	*PHLDA1*	Hom	chr12:76424937	c.582_584delGCA	p.Gln204del	Benign
25	*ENAM*	Hom	chr4:71509086	c.1943T>C	p.Ile648Thr	Benign
25	*ENAM*	Hom	chr4:71509431	c.2288G>A	p.Arg763Gln	Benign
26	*AHNAK2*	Hom	chr14:105415352	c.6436C>G	p.Leu2146Val	Benign
27	*ARHGEF17*	Hom	chr11:73020375	c.703_705dup	p.Ser235dup	Benign
27	*RELT*	Hom	chr11:73100232	c.45+6C>T	-	Benign
27	*ZNF485*	Hom	chr10:44104101	c.64C>T	p.Arg22Trp	Benign
27	*CFAP61*	Hom	chr20:20079360	c.761A>G	p.His254Arg	Benign
27	*LATS2*	Hom	chr13:21563311	c.608C>T	p.Ala203Val	Benign
27	*ASPN*	Hom	chr9:95237024	c.153_155delTGA	p.Asp51del	Benign
27	*MORC1*	Hom	chr3:108780831	c.966+4C>T	-	Benign
27	*CHST9*	Hom	chr18:24497190	c.365G>A	p.Ser122Asn	Benign
27	*ECE2*	Hom	chr3:183976241	c.646A>G	p.Lys216Glu	Benign
27	*HIST1H4G*	Hom	chr6:26247198	c.8T>C	p.Val3Ala	Benign
27	*LAMTOR5*	Hom	chr1:110950277	c.212C>T	p.Pro71Leu	Benign
27	*POU4F2*	Hom	chr4:147560457	c.198_200dup	p.Gly68dup	Benign
28	*PTGR1*	Hom	chr9:114355266	c.153_154delTT	-	Uncertain significance

28	*ABI3BP*	Hom	chr3:100570787	c.1160_1161insTTT	-	Uncertain significance
28	*BUD13*	Hom	chr11:116633322	c.983G>A	p.Arg328Gln	Likely benign
28	*SLC12A4*	Hom	chr16:68002495	c.51_62delGACAGCGGCGGG	p.Gly17_Gly21del	Benign
28	*TMEM200C*	Hom	chr18:5891041	c.1022C>T	p.Ala341Val	Benign
28	*UGT2B11*	Hom	chr4:70079975	c.466T>C	p.Cys156Arg	Benign
28	*FBN3*	Hom	chr19:8168545	c.4840G>A	p.Gly1614Ser	Benign
28	*OR51A2*	Hom	chr11:4976447	c.497G>C	p.Arg166Thr	Benign
28	*FRAT2*	Hom	chr10:99094083	c.247G>A	p.Ala83Thr	Benign
28	*TUBGCP6*	Hom	chr22:50656428	c.5287C>T	p.Arg1763Trp	Benign
29	*FZD6*	Hom	chr8:104312432	c.1A>G	p.Met1?	Likely pathogenic
29	*TOP2B*	Hom	chr3:25679805	c.396-8_396-5del	-	Uncertain significance
29	*RP1L1*	Hom	chr8:10469233	c.2375T>C	p.Leu792Pro	Benign
29	*ENPP3*	Hom	chr6:132061420	c.2357G>A	p.Ser786Asn	Benign
29	*HRH4*	Hom	chr18:22056766	c.413C>T	p.Ala138Val	Benign
29	*MEGF6*	Hom	chr1:3410973	c.4091G>A	p.Arg1364His	Benign
29	*WEE2*	Hom	chr7:141429371	c.1576T>G	p.Tyr526Asp	Benign
29	*MEGF6*	Hom	chr1:3424388	c.1760C>T	p.Pro587Leu	Benign
29	*TRIM5*	Hom	chr11:5686266	c.1255C>T	p.His419Tyr	Benign
29	*TOP2A*	Hom	chr17:38557317	c.2449T>G	p.Leu817Val	Benign

29	*DYNC2LI1*	Hom	chr2:44028013	c.691A>T	p.Ile231Leu	Benign
29	*DSE*	Hom	chr6:116720487	c.74C>T	p.Thr25Ile	Benign
29	*RP1L1*	Hom	chr8:10470068	c.1540G>A	p.Gly514Ser	Benign
29	*MGME1*	Hom	chr20:17950545	c.43A>T	p.Ser15Cys	Benign
29	*ACSBG2*	Hom	chr19:6187686	c.1757G>A	p.Gly586Asp	Benign
29	*MEGF6*	Hom	chr1:3410410	c.4312G>A	p.Gly1438Arg	Benign
29	*HRH4*	Hom	chr18:22056970	c.617A>G	p.His206Arg	Benign
29	*RP1L1*	Hom	chr8:10466482	c.5126C>T	p.Ala1709Val	Benign
29	*ASPN*	Hom	chr9:95237024	c.153_155delTGA	p.Asp51del	Benign
29	*WEE2*	Hom	chr7:141422926	c.881-8T>C	-	Benign
29	*RP1L1*	Hom	chr8:10465097	c.6511G>A	p.Glu2171Lys	Benign
29	*TCHH*	Hom	chr1:152083325	c.2368T>A	p.Leu790Met	Benign
29	*OR51I2*	Hom	chr11:5475431	c.714_715insCA	p.Leu238_Asn239fs	Uncertain significance
29	*DEFB116*	Hom	chr20:29891242	c.82T>A	p.Ser28Thr	Benign
29	*EFNA3*	Hom	chr1:155058663	c.568G>A	p.Val190Met	Benign
29	*WTIP*	Hom	chr19:34973087	c.208G>A	p.Glu70Lys	Benign
29	*CACNA1S*	Hom	chr1:201052310	c.1373T>A	p.Leu458His	Benign
29	*ACSBG2*	Hom	chr19:6187800	c.1871G>A	p.Arg624Lys	Benign
29	*ACSBG2*	Hom	chr19:6187805	c.1876G>C	p.Glu626Gln	Benign

29	*MEGF6*	Hom	chr1:3417211	c.2693C>T	p.Pro898Leu	Benign
29	*ACSBG2*	Hom	chr19:6187680	c.1751G>A	p.Gly584Asp	Benign
29	*MROH2A*	Hom	chr2:234704607	c.1060-6C>T	-	Benign
30	*ECPAS*	Hom	chr9:114184259	c.1931C>G	p.Thr644Ser	Uncertain significance

Table S2. Pathogenic or likely pathogenic variants in the OFC-related morphogenic processes or pathways identified in 30 hereditary families.

Family ID	Gene	Pathway	Zygosity	Genomic position	cDNA change	Protein change	ACMG category	Evidence
12	*PTCH1*	HH	Het	chr9:98241322	c.1175C>T	p.A392V	Pathogenic	PS1 + PS3 + PM2 + PP3 + PP5
16	*PTCH1*	HH	Het	chr9:98221936	c.2833C>T	p.R945*	Pathogenic	PVS1 + PS3 + PM2
21	*GLI2*	HH	Het	chr2:121748112	c.4622C>A	p.S1541Y	Likely pathogenic	PS3 + PP1 + PP3 + PP5
21	*GLI2*	HH	Het	chr2:121747840	c.4350G>T	p.Q1450H	Likely pathogenic	PS3 + PP1 + PP3 + PP5
21	*GLI2*	HH	Het	chr2:121746174	c.2684C>T	p.A895V	Likely pathogenic	PS3 + PM2 + PP1 + PP3 + PP5
18	*IRF6*	Epithelial-related	Het	chr1:209974733	c.26G>A	p.R9Q	Pathogenic	PS3 + PM1 + PM2 + PM5 + PP5
9	*PLEKHA5*	Epithelial-related	Het	chr12:19406939	c.293T>C	p.I98T	Likely pathogenic	PS3 + PM2
3	*CREBBP*	TGF-β	Het	chr16:3819324	c.2911A>G	p.R971G	Likely pathogenic	PS3 + PM2
29	*FZD6*	WNT	Hom	chr8:104312432	c.1A>G	p.M1?	Likely pathogenic	PVS1 + PM4 + PP1

Table S3. Summary of clinical information of the 30 hereditary families with NSOFC.

Family ID	Inheritance model	Ethnic group	Sex and NSOFC phenotypes		
			D1 (proband)	D2	D3
1	AD	Han	M; RCLP	M; RCL + MCP	/
2	AD	Han	M; BCL + MCP	F; BCL + MCP	/
3	AD	Han	M; RCL + CP + RCA	M; LCL + LCA	/
4	AD	Han	F; LCL + LCA	F; MCP	/
5	AD	Han	M; MCP	F; CP	/
6	AD	Han	M; LCLP	F; CP	/
7	AD	Han	M; LCLP	M; RCL	/
8	AD	Han	M; RCLP	M; BCLP	/
9	AD	Han	M; BCL	M; RCL	/
10	AD	Han	M; CLP	F; CLP	/
11	AD	Han	F; CP	F; CP	/
12	AD	Han	F; RCLP + RCA	F; RCLP + RCA	/
13	AD	Han	F; BCL	M; CLP	/
14	AD	Han	F; LCL + LCA	M; LCL + CP	/
15	AD	Han	M; CP	F; CP	F; CP
16	AD	Han	M; BCLP	F; LCL + LCA	F; high palate arch
17	AD	Han	M; CP	F; CP	/
18	AD	Han	M; LCL	F; CP	/
19	AD	Han	M; LCL + CP	M; LCL + CP	/
20	AD	Han	F; RCLP	F; CLP	/
21	AD	Hui	M; LCLP	F; LCL	/

22	AD	Uighur	F; RCLP	M; RCL	/
23	AD	Uighur	F; BCLP	M; LCL	/
24	AR	Han	F; BCLP	F; RCL + RCA	/
25	AR	Han	F; BCL	M; BCLP	/
26	AR	Han	M; LCLP	F; LCL	/
27	AR	Uighur	F; LCLP	/	/
28	AR	Kazak	M; LCL	/	/
29	AR	Uighur	F; LCLP	/	/
30	AR	Han	M; LCLP	F; LCL	/

AD, Autosomal dominant; AR, Autosomal recessive.

M, Male; F, Female.

R, Right; L, Left; M, Median.

CLP, Cleft lip and palate; CL, Cleft lip; CP, Cleft palate only; CA, Alveolar bone cleft.

Table S4. Summary of the sequencing and alignment data of WES.

Family ID	Subject ID	Total effective reads	Total effective yield (Gb)	Average sequencing depth on target (X)	Q20 (%)	Q30 (%)	Mapping rate on genome (%)	Coverage of exome (%)	Fraction of target covered ≥ 4x (%)	Fraction of target covered ≥ 20x (%)
1	D1	216,975,801	21.07	267.01	97.32	90.41	99.94	99.83	99.6	98.4
1	D2	254,617,437	24.57	221.88	97.66	91.17	99.95	99.92	99.72	97.8
1	C1	296,117,617	28.63	277.19	97.5	90.59	99.95	99.79	99.59	98.28
2	D1	211,014,846	20.51	265.7	97.36	90.09	99.96	99.78	99.53	98.07
2	D2	218,781,139	21.2	195.4	97.82	91.26	99.94	99.75	99.46	97.47
2	C1	266,358,402	25.75	212.59	98.1	92.12	99.94	99.78	99.53	97.46
2	C2	213,089,396	20.7	255.54	97.09	89.82	99.95	99.83	99.58	98.35
3	D1	233,372,429	22.55	197.45	97.81	91.25	99.93	99.95	99.7	97.49
3	D2	285,400,457	27.59	237.26	97.58	90.83	99.94	99.95	99.79	98.3
3	C1	214,009,039	20.81	271.81	97.49	90.63	99.96	99.63	99.4	98.17
4	D1	239,341,604	23.24	293.33	97.63	91.07	99.96	99.68	99.43	98.29
4	D2	280,205,661	27.05	239.72	97.69	90.82	99.95	99.78	99.58	97.72
4	C1	281,315,152	27.19	229.88	97.71	91.23	99.95	99.93	99.75	98.19
4	C2	245,518,509	23.78	207.63	97.52	91.03	99.94	99.91	99.64	97.46
5	D1	301,362,126	29.18	268.94	97.46	90.81	99.94	99.92	99.73	98.26
5	D2	280,712,464	27.14	218.38	97.63	91.27	99.92	99.79	99.59	97.65
5	C1	279,132,183	27.1	312.93	97.43	90.81	99.91	99.88	99.65	98.62
5	C2	251,529,546	24.32	203.46	97.13	89.91	99.94	99.8	99.59	97.64
6	D1	223,975,126	21.56	180.42	95.17	87.83	99.92	99.89	99.52	96.45

6	D2	249,743,630	23.98	247.22	94.29	85.97	99.95	99.73	99.37	97.45
6	C1	268,096,165	25.96	225.46	97.97	91.65	99.91	99.92	99.7	98
7	D1	232,906,537	22.48	256.43	94.33	85.87	99.95	99.85	99.51	97.74
7	D2	248,916,022	23.94	168.13	95.54	88.62	99.9	99.92	99.62	96.46
7	C1	237,523,986	23.04	279.44	97.62	91.19	99.97	99.72	99.43	97.33
8	D1	206,883,543	19.94	184.82	95.53	88.55	99.93	99.9	99.54	96.9
8	D2	200,433,869	19.32	220.1	94.49	86.19	99.96	99.86	99.5	97.38
8	C1	271,661,189	26.32	319.01	97.59	91.06	99.96	99.73	99.46	97.88
9	D1	228,727,084	21.98	185.51	94.72	87	99.92	99.89	99.49	96.7
9	D2	213,354,405	20.57	202.5	95.53	88.63	99.93	99.87	99.52	97
9	C1	276,844,038	26.78	204.39	97.83	91.69	99.93	99.76	99.55	97.38
10	D1	214,053,274	20.67	234.15	94.17	85.48	99.96	99.69	99.28	97.11
10	D2	269,757,536	25.98	220.48	97.01	90.31	99.94	99.92	99.69	97.43
10	C1	260,244,405	25.18	223.95	97.73	91.31	99.94	99.93	99.69	97.87
11	D1	267,457,362	25.93	242.32	97.2	90.74	99.95	99.75	99.47	97.58
11	D2	287,286,752	27.89	239.74	97.18	90.6	99.95	99.76	99.44	97.31
11	C1	224,846,867	21.81	253.24	97.51	90.7	99.95	99.88	99.59	98.07
12	D1	216,595,638	21.05	257.87	97.58	91.54	99.98	99.69	99.39	97.81
12	D2	303,712,468	29.45	249.21	97.1	90.42	99.95	99.77	99.52	97.56
12	C1	238,725,763	23.21	302.71	97.5	90.89	99.97	99.79	99.56	98.29
13	D1	225,876,745	21.98	219.37	97.55	91.33	99.96	99.72	99.34	97.07
13	D2	192,612,326	18.68	170.59	97.28	90.94	99.95	99.9	99.48	96.27
13	C1	232,732,578	22.64	286.15	98.05	92.06	99.94	99.65	99.39	98.15
14	D1	233,095,285	22.36	198.17	93.7	84.67	99.95	99.74	99.29	96.57

14	D2	227,382,876	21.86	281.9	94.05	85.48	99.96	99.79	99.5	97.93
14	C1	215,942,792	20.99	258.45	98	91.97	99.93	99.66	99.37	97.59
15	D1	263,146,896	25.51	217.85	97.13	90.51	99.94	99.93	99.64	97.27
15	D2	281,709,610	27.22	248.01	97.46	91.2	99.95	99.8	99.59	97.87
15	D3	248,237,791	24.13	303.52	96.96	90.22	99.97	99.7	99.42	97.98
15	C1	238,185,906	23.16	282.3	97.7	91.4	99.96	99.87	99.62	98.48
16	D1	254,516,026	24.45	185.9	96.01	89.36	99.93	99.94	99.65	96.73
16	D2	273,926,560	26.56	316.41	97.64	91.74	99.97	99.74	99.49	98.3
16	D3	231,256,741	22.47	288.65	97.13	89.89	99.95	99.67	99.42	98.27
16	C1	283,268,430	27.44	322.85	97.81	91.46	99.94	99.92	99.67	98.42
16	C2	220,095,079	21.35	214.99	97.89	91.9	99.95	99.9	99.6	97.62
17	D1	289,899,890	28.02	229.59	97.88	92.24	99.95	99.95	99.77	97.57
17	D2	254,690,459	24.51	221.74	95.88	89.13	99.97	99.76	99.47	97.28
17	C1	143,035,542	13.87	146.57	96.84	88.7	99.9	99.84	99.47	96.46
18	D1	241,444,709	23.16	204.14	95.52	88.63	99.93	99.92	99.61	95.86
18	D2	243,347,126	23.31	185.58	95.17	87.93	99.93	99.77	99.51	96.86
18	C1	250,559,191	24.38	310.77	97.26	89.91	99.96	99.81	99.59	98.61
19	D1	325,356,028	31.43	247.61	97.08	90.72	99.93	99.95	99.8	98.19
19	D2	244,088,483	23.73	297.37	97.52	91.35	99.97	99.84	99.6	98.5
19	C1	273,498,301	26.53	268.09	96.87	89.1	99.94	99.78	99.54	98.36
20	D1	243,508,612	23.41	178.24	95.7	88.72	99.94	99.75	99.41	96.34
20	D2	227,622,240	22.13	224.05	97.62	91.55	99.96	99.74	99.39	97.26
20	C1	236,777,794	22.95	207.96	97.07	89.23	99.94	99.92	99.62	97.54
21	D1	237,728,368	23.06	227.68	97.93	92.13	99.95	99.91	99.59	97.53

21	D2	247,675,800	24.07	268.58	97.9	92.02	99.97	99.73	99.41	97.82
21	C1	193,303,580	18.78	211.1	97.36	90.02	99.95	99.72	99.38	97.42
21	C2	188,018,514	18.25	208.74	97.69	91.01	99.95	99.87	99.58	97.74
22	D1	247,010,070	24.04	289.31	97.61	91.54	99.97	99.69	99.36	97.76
22	D2	224,143,893	21.61	246.56	94.2	85.47	99.96	99.72	99.35	97.48
22	C1	254,345,860	24.62	272.41	97.54	91.13	99.92	99.91	99.69	98.34
22	C2	204,288,542	19.88	254.53	97.46	90.3	99.95	99.78	99.47	97.7
23	D1	254,417,160	24.7	253.89	97.6	91.55	99.96	99.75	99.39	97.43
23	D2	276,389,245	26.66	247.93	97.44	91.1	99.95	99.93	99.71	97.79
23	C1	265,702,969	25.78	319.24	98.32	92.52	99.96	99.71	99.46	98.34
24	D1	202,108,996	19.44	181.27	95.44	88.4	99.95	99.9	99.54	96.76
24	D2	268,223,864	25.78	203.27	95.26	88.04	99.94	99.8	99.52	97.09
24	C1	186,873,674	18.17	236.01	96.68	88.3	99.92	99.78	99.54	98.1
25	D1	292,953,863	28.09	220.07	95.96	89.26	99.94	99.78	99.57	97.13
25	D2	218,125,597	20.82	160.1	96.03	89.4	99.94	99.92	99.58	95.31
25	C1	215,344,569	20.84	178.96	97.35	89.97	99.91	99.92	99.64	97.21
26	D1	233,962,692	22.37	198.64	94.16	85.71	99.92	99.91	99.54	97.01
26	D2	254,807,532	24.76	270.4	97.52	91.28	99.96	99.72	99.41	97.77
26	C1	311,003,455	30.06	192.04	97.96	92.04	99.91	99.94	99.75	97.61
27	D1	220,271,327	21.41	270.15	97.51	91.33	99.98	99.67	99.33	97.7
27	C1	285,443,375	27.61	258.3	98.27	92.27	99.94	99.79	99.56	97.89
27	C2	239,677,586	23.25	271.76	98.25	92.22	99.96	99.88	99.61	98.24
28	D1	203,788,001	19.81	237.99	97.98	92.28	99.97	99.82	99.5	97.81
28	C1	266,907,167	25.85	328.89	97.89	91.74	99.95	99.71	99.47	98.52

28	C2	223,759,607	21.78	287.77	97.78	91.33	99.95	99.8	99.59	98.53
29	D1	256,432,597	24.91	256.79	98.01	92.31	99.96	99.76	99.43	97.71
29	C1	236,529,203	22.88	206.41	97.96	91.7	99.93	99.77	99.51	97.25
29	C2	235,180,362	22.77	206.5	97.88	91.49	99.93	99.94	99.69	97.53
30	D1	87,994,638	11.98	101.71	98.32	95.64	98.73	99.9	99.7	97.7
30	D2	72,780,156	9.91	84.01	98.33	95.7	98.75	99.8	99.5	96.6
30	C1	87,461,858	11.9	102.09	98.55	95.69	98.82	99.9	99.7	97.7
	**Average**	245,519,312	23.37	235.25	97	90.35	99.91	99.82	99.54	97.61

Table S5. Summary of the identified variants by WES.

Family ID	Subject ID	Total Variants	Homozygotes	Heterozygotes	Exonic	Intronic	Intergenic	Upstream	Downstream	Splicing	Missense	Frameshift
1	D1	113,317	50,076	63,241	30,804	75,039	2,990	2,729	1,755	148	10,256	306
1	D2	127,661	58,342	69,319	31,496	87,353	3,410	3,331	2,071	147	10,242	292
1	C1	132,466	58,815	73,651	32,139	91,256	3,405	3,420	2,246	149	10,410	298
2	D1	117,327	51,993	65,334	31,101	78,819	2,919	2,654	1,834	144	10,380	284
2	D2	126,253	57,900	68,353	31,444	86,293	3,385	3,086	2,045	147	10,334	299
2	C1	130,836	58,550	72,286	31,387	90,537	3,613	3,183	2,116	143	10,172	299
2	C2	119,816	53,142	66,674	31,055	80,779	3,179	2,871	1,932	143	10,276	312
3	D1	128,853	58,024	70,829	31,635	88,419	3,384	3,324	2,091	147	10,337	299
3	D2	134,914	60,652	74,262	31,802	93,780	3,675	3,469	2,188	154	10,296	306
3	C1	117,077	50,491	66,586	30,967	78,539	3,005	2,746	1,820	144	10,297	315
4	D1	118,813	51,823	66,990	31,231	79,591	3,141	2,916	1,934	164	10,274	304
4	D2	129,594	58,360	71,234	31,396	89,133	3,440	3,458	2,167	165	10,108	292
4	C1	132,496	59,616	72,880	32,011	91,434	3,425	3,396	2,230	152	10,451	305
4	C2	128,352	59,222	69,130	31,501	88,130	3,403	3,188	2,130	154	10,298	289
5	D1	130,013	58,674	71,339	31,675	89,712	3,252	3,215	2,159	156	10,351	310
5	D2	131,456	59,130	72,326	31,843	90,764	3,312	3,366	2,171	152	10,402	305
5	C1	123,850	55,289	68,561	31,226	84,400	3,156	3,059	2,009	157	10,250	314
5	C2	131,195	58,525	72,670	31,463	90,783	3,471	3,258	2,220	142	10,277	297
6	D1	122,913	56,338	66,575	31,393	83,414	3,180	2,872	2,054	144	10,482	281
6	D2	122,533	54,379	68,154	31,265	83,411	3,106	2,778	1,973	148	10,484	299
6	C1	130,716	59,025	71,691	31,783	90,071	3,341	3,343	2,178	143	10,320	298

7	D1	118,152	54,068	64,084	30,775	79,777	3,064	2,608	1,928	150	10,238	308
7	D2	126,012	58,752	67,260	31,233	86,174	3,396	3,107	2,102	136	10,262	295
7	C1	120,860	53,361	67,499	31,582	81,110	3,027	3,137	2,004	146	10,355	299
8	D1	118,623	54,222	64,401	31,081	79,717	3,179	2,711	1,935	149	10,351	296
8	D2	115,152	51,837	63,315	30,565	77,058	3,173	2,541	1,815	147	10,199	297
8	C1	117,656	50,746	66,910	31,325	78,379	3,088	3,008	1,856	153	10,344	301
9	D1	124,901	57,736	67,165	31,314	85,585	3,270	2,724	2,008	132	10,316	302
9	D2	116,930	53,756	63,174	30,990	78,298	3,230	2,567	1,845	146	10,389	305
9	C1	134,862	59,688	75,174	32,178	93,502	3,457	3,443	2,282	138	10,419	310
10	D1	114,762	51,610	63,152	30,788	76,604	3,107	2,478	1,785	141	10,403	300
10	D2	128,613	58,053	70,560	31,778	88,104	3,339	3,233	2,159	146	10,462	304
10	C1	130,530	59,079	71,451	31,558	90,055	3,326	3,380	2,211	138	10,343	300
11	D1	127,397	57,429	69,968	31,604	87,310	3,379	3,046	2,058	150	10,422	293
11	D2	131,201	58,459	72,742	31,709	90,824	3,451	3,067	2,150	143	10,341	282
11	C1	118,684	52,412	66,272	30,958	79,568	3,253	2,962	1,943	140	10,265	291
12	D1	117,208	52,221	64,987	30,736	78,879	2,980	2,748	1,865	147	10,241	279
12	D2	132,978	59,064	73,914	31,735	92,667	3,107	3,305	2,164	151	10,393	283
12	C1	114,309	50,947	63,362	30,640	76,131	2,989	2,741	1,808	144	10,201	297
13	D1	123,687	56,123	67,564	31,244	84,733	3,031	2,741	1,938	133	10,424	304
13	D2	119,213	55,204	64,009	30,750	80,683	3,229	2,687	1,864	138	10,331	289
13	C1	115,252	50,323	64,929	31,310	76,500	2,924	2,763	1,755	145	10,514	316
14	D1	129,600	56,179	73,421	31,506	89,663	3,496	2,862	2,073	134	10,332	271
14	D2	117,957	51,929	66,028	30,956	79,675	2,896	2,588	1,842	127	10,272	288
14	C1	113,159	49,889	63,270	31,035	74,579	3,112	2,735	1,698	150	10,334	310

15	D1	128,610	59,031	69,579	31,426	88,592	3,374	3,075	2,143	153	10,300	290
15	D2	130,144	58,477	71,667	31,758	89,391	3,550	3,305	2,140	145	10,320	289
15	D3	119,214	52,463	66,751	30,878	80,530	3,127	2,753	1,926	147	10,170	291
15	C1	121,685	54,154	67,531	31,297	82,225	3,163	3,032	1,968	153	10,341	298
16	D1	125,407	57,864	67,543	31,120	85,972	3,248	3,048	2,019	150	10,220	291
16	D2	119,543	52,244	67,299	31,142	80,581	2,966	2,930	1,924	146	10,346	296
16	D3	114,593	49,659	64,934	30,778	76,338	2,985	2,682	1,810	134	10,213	294
16	C1	121,304	53,068	68,236	31,157	81,981	3,164	3,052	1,950	147	10,268	298
16	C2	122,424	55,771	66,653	30,973	83,394	3,202	2,918	1,937	145	10,261	281
17	D1	130,107	60,730	69,377	31,448	89,539	3,420	3,500	2,200	145	10,183	316
17	D2	126,768	56,866	69,902	31,484	86,653	3,477	3,032	2,122	146	10,391	291
17	C1	116,659	53,775	62,884	31,140	78,046	2,947	2,683	1,843	144	10,478	280
18	D1	120,902	55,607	65,295	31,352	81,213	3,222	3,160	1,955	136	10,431	306
18	D2	124,129	57,113	67,016	31,489	84,134	3,438	3,052	2,016	139	10,514	307
18	C1	123,406	53,897	69,509	31,403	83,854	3,182	2,975	1,992	154	10,262	312
19	D1	135,611	59,414	76,197	32,307	94,305	3,300	3,455	2,244	142	10,586	300
19	D2	118,667	52,462	66,205	31,120	79,682	3,131	2,785	1,949	144	10,361	301
19	C1	131,200	57,405	73,795	32,040	90,329	3,349	3,328	2,154	148	10,509	295
20	D1	128,254	56,967	71,287	31,625	88,282	3,171	3,056	2,120	153	10,476	279
20	D2	125,541	56,313	69,228	31,318	86,254	2,992	2,893	2,084	142	10,300	311
20	C1	132,582	59,229	73,353	31,855	92,081	3,236	3,201	2,209	141	10,477	311
21	D1	124,813	55,513	69,300	31,577	84,918	3,257	3,017	2,044	150	10,361	312
21	D2	124,365	53,907	70,458	31,543	84,648	3,099	3,022	2,053	149	10,284	301
21	C1	118,331	52,040	66,291	31,228	79,203	3,217	2,849	1,834	147	10,379	299

21	C2	119,723	52,380	67,343	31,559	80,210	3,145	2,940	1,869	149	10,468	302
22	D1	124,710	48,954	75,756	31,838	84,974	3,175	2,723	2,000	152	10,429	293
22	D2	118,389	47,754	70,635	31,488	79,302	3,220	2,527	1,852	139	10,542	287
22	C1	121,753	49,704	72,049	31,893	81,710	3,115	3,018	2,017	160	10,473	302
22	C2	118,700	47,729	70,971	31,396	79,833	3,022	2,596	1,853	150	10,389	304
23	D1	126,741	50,782	75,959	31,991	86,371	3,356	3,005	2,018	137	10,639	295
23	D2	130,110	53,539	76,571	32,047	89,326	3,267	3,308	2,162	151	10,429	305
23	C1	116,350	47,035	69,315	31,248	77,191	3,133	2,907	1,871	144	10,450	299
24	D1	118,672	54,160	64,512	31,093	79,731	3,219	2,749	1,880	140	10,396	309
24	D2	126,563	57,268	69,295	31,337	86,793	3,216	3,111	2,106	139	10,335	302
24	C1	117,834	52,520	65,314	30,984	79,144	3,079	2,779	1,848	143	10,293	309
25	D1	129,204	57,499	71,705	31,672	88,756	3,379	3,280	2,117	151	10,252	265
25	D2	122,729	56,804	65,925	31,484	82,822	3,392	3,069	1,962	151	10,400	276
25	C1	127,440	57,812	69,628	31,577	87,164	3,428	3,189	2,082	142	10,492	307
26	D1	122,589	56,155	66,434	31,022	83,729	3,104	2,803	1,931	148	10,343	290
26	D2	122,880	54,665	68,215	31,115	83,592	3,196	2,899	2,078	149	10,322	304
26	C1	136,065	60,981	75,084	31,700	94,986	3,640	3,484	2,255	153	10,259	298
27	D1	118,273	45,724	72,549	31,456	79,253	2,961	2,678	1,925	153	10,485	298
27	C1	128,572	54,228	74,344	31,960	87,876	3,262	3,303	2,171	145	10,508	298
27	C2	118,459	48,185	70,274	31,437	79,370	2,903	2,886	1,863	139	10,462	291
28	D1	113,747	58,501	55,246	29,452	76,744	3,137	2,672	1,742	148	9,599	306
28	C1	117,284	48,783	68,501	31,450	77,849	3,281	2,915	1,789	156	10,427	300
28	C2	115,243	47,228	68,015	30,947	76,783	2,935	2,737	1,841	138	10,337	295
29	D1	125,777	50,782	74,995	31,800	85,504	3,360	3,021	2,092	142	10,490	318

29	C1	126,223	52,047	74,176	31,616	85,778	3,528	3,241	2,060	144	10,373	311
29	C2	125,519	55,863	69,656	31,541	85,257	3,326	3,255	2,140	139	10,354	312
30	D1	73,436	31,106	42,330	20,831	21,151	3,469	1,103	1,371	75	9,448	121
30	D2	73,357	30,768	42,589	20,793	21,126	3,509	1,045	1,411	77	9,442	138
30	C1	73,051	31,101	41,950	20,623	21,082	3,431	1,040	1,375	78	9,316	115
	**Average**	122,301	54,120	68,182	31,065	82,310	3,236	2,943	1,990	144	10,323	293
